# Problematic Ar^F^–Alkynyl Coupling with Fluorinated Aryls. From Partial Success with Alkynyl Stannanes to Efficient Solutions via Mechanistic Understanding of the Hidden Complexity

**DOI:** 10.1021/jacs.2c10842

**Published:** 2022-12-21

**Authors:** Guillermo Marcos-Ayuso, Marconi N. Peñas-Defrutos, Ana M. Gallego, Max García-Melchor, Jesús M. Martínez-Ilarduya, Pablo Espinet

**Affiliations:** † IU CINQUIMA/Química Inorgánica, Facultad de Ciencias, Universidad de Valladolid, Valladolid E-47071, Spain; ‡ School of Chemistry, CRANN and AMBER Research Centres, Trinity College Dublin, College Green, Dublin 2, Ireland

## Abstract

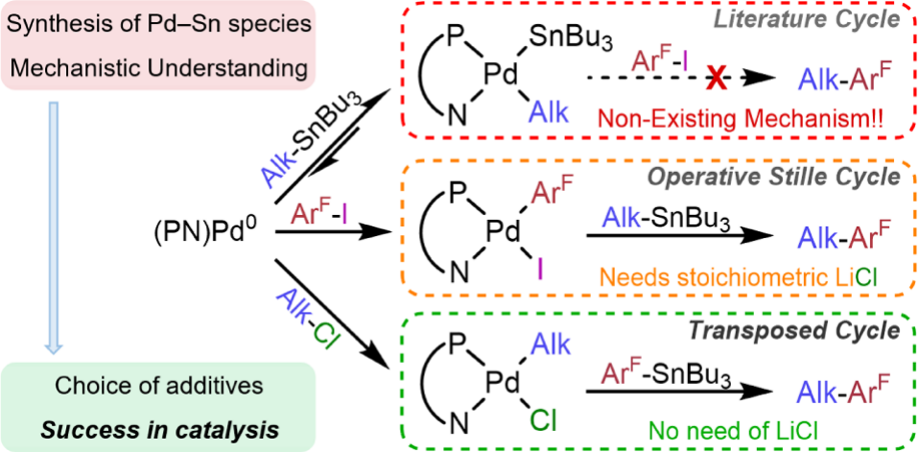

The synthesis of aryl–alkynyl compounds is usually achieved via Sonogashira catalysis, but this is inefficient for fluorinated aryls. An alternative method reported by Shirakawa and Hiyama, using alkynylstannanes and hemilabile PN ligands, works apparently fine for conventional aryls, but it is also poor for fluorinated aryls. The revision of the unusual literature cycle reveals the existence and nature of unreported byproducts and uncovers coexisting cycles and other aspects that explain the reasons for the conflict. This knowledge provides a full understanding of the real complexity of these aryl/alkynylstannane systems and the deviations of their evolution from that of a classic Stille process, providing the clues to design several very efficient alternatives for the catalytic synthesis of the desired Ar^F^–alkynyl compounds in almost quantitative yield. The same protocols are also very efficient for the catalytic synthesis of alkynyl–alkynyl’ hetero- and homocoupling.

## Introduction

**Scheme 1:**
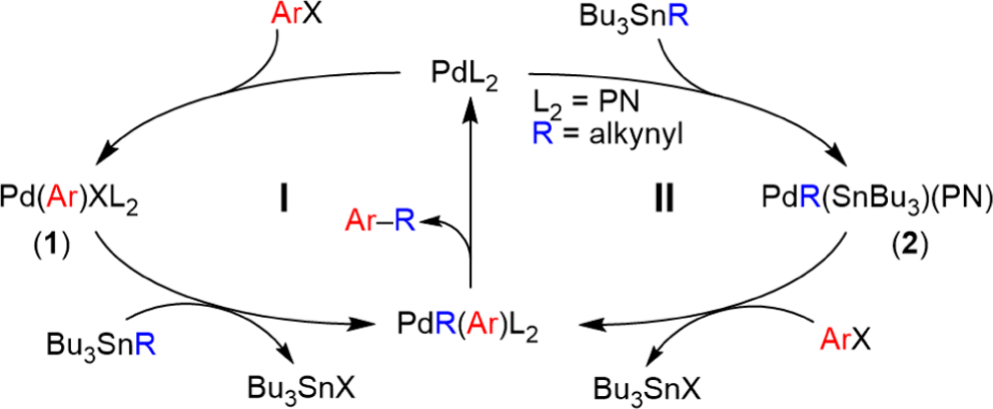
Classic Stille Cycle (I) and the Alternative Pathway (II) Proposed by Shirakawa and Hiyama for Aryl–Alkynyl Coupling, Represented for a PN Ligand

Since its discovery, Stille catalysis has proven to be an excellent method for C–C coupling.^1^ Although less frequently used nowadays, it has an advantage that it can be applied to reagent-bearing groups that would not stand the reaction conditions of other name reactions. The mechanisms of the different steps of the classic cycle (Scheme 1, cycle **I**), namely, Ar–X oxidative addition, Bu_3_SnR transmetalation, and Ar–R reductive elimination, have been extensively studied.^2^

About 20 years ago, Shirakawa and Hiyama proposed that a different mechanism operated for the case of alkynyl stannanes (Scheme 1, cycle **II**, R = alkynyl, shortened as Alk from now on) and was particularly efficient with iminophosphine or aminophosphine chelating ligands (PN), but inefficient with PPh_3_ or diphosphines. In this cycle, the roles of the electrophile and the nucleophile on Pd, are altered relative to cycle **I**: instead of the Ar–X electrophile, the alkynyl stannane acts as the oxidant on Pd^0^(PN), formed in situ from the precatalyst (μ-Cl)_2_[Pd(π-allyl)]_2_ and an iminophosphine or aminophosphine, to give [Pd(Alk)(SnBu_3_)(PN)] (**2**).^3^ Then, Ar–X produces an Ar/SnBu_3_ exchange by an unexplained mechanism.^4,5^ The formation of **2** with PN ligands was experimentally ascertained by its NMR observation,^6^ but the operativity of the subsequent Ar/SnBu_3_ exchange proposed has never been demonstrated nor denied. Crociani et al. reported that using [Pd^0^(PN)(η^2^-dimethylfumarate)] as catalyst complex **2** was not observed and the classic cycle **I**, initiated by oxidative addition with Ar–X, was operating.^7^ Our interest in C–C couplings involving fluorinated aryls led us in the past to test the Sonogashira catalysis, the classic method for Ar–Alk coupling, but we found in preliminary experiments that, for Ar^F^ = C_6_F_3_Cl_2_-3,5, this process was quite inefficient: from (C_6_F_3_Cl_2_)–I and Bu_3_Sn–C≡C–Ph ([PdCl_2_(PPh_3_)_2_]/CuI, NEt_3_, 80 °C, dioxane, 24 h), it produced 90% conversion, but only 2% was the desired product and 88% was (C_6_F_3_Cl_2_)–H, confirming that, as it often happens, fluorinated aryls are a different challenge.^8^

We tried then the Stille reaction with alkynyl stannanes.^9^ The 1:1 coupling of Ar^F^–I with PhC≡CSnBu_3_, in tetrahydrofuran (THF) at 50 °C, catalyzed by 5% of [Pd(Ar^F^)I(PPh_3_)_2_] (either *cis* or *trans*) showed that the catalysis follows cycle **I** but is very inefficient, producing less than 5% of Ar^F^–Alk in 3 h. The catalytic problem was identified with the fact that the *trans*-to-*cis* isomerization, required to give coupling, was extremely slow, and eventually, *trans*-[Pd(Alk)(Ar^F^)(PPh_3_)_2_] became a catalyst trap. Curiously, the nonanalyzed Shirakawa’s results reported for conventional aryls and 2 PPh_3_ instead of PN were even worse (about 1% cross-coupling), as if their reaction, like ours, failed to be efficient in cycle **I** with PPh_3_.

The positive results reported by Shirakawa and Hiyama for Ar–Alk coupling of conventional aryls, using PN ligands and supposedly following cycle **II**, provided yields in the range of 86–93% (18–29 h in THF at 50 °C, 5 mol % of the (μ-Cl)_2_[Pd(π-allyl)]_2_ precatalyst, i.e., 10% Pd).^5^ They looked for an attractive alternative to an inefficient Sonogashira. However, when we checked the reaction of IC_6_F_3_Cl_2_-3,5 with Bu_3_Sn–C≡C–Ph using [Pd(Alk)(SnBu_3_)(PN)] (**2**) (10% **2**, in THF, 50 °C, 24 h) as catalyst, the result was not only somewhat disappointing but also puzzling: 50% C_6_F_3_Cl_2_–Alk, 28% C_6_F_3_Cl_2_–SnBu_3_ and 22% C_6_F_3_Cl_2_–H.

Considering all the apparently contradictory data of the previous information, this study has two targets: (i) to find out what is exactly going on in the reactions involving aryl electrophiles and alkynylstannane nucleophiles and why they are inefficient for fluorinated aryls and (ii) to develop efficient catalytic protocols for catalytic synthesis of Ar^F^–Alk compounds, potential precursors of many other fluorinated species.

## Results and Discussion

### Section A: Mechanistic Stoichiometric Studies

**Scheme 2:**
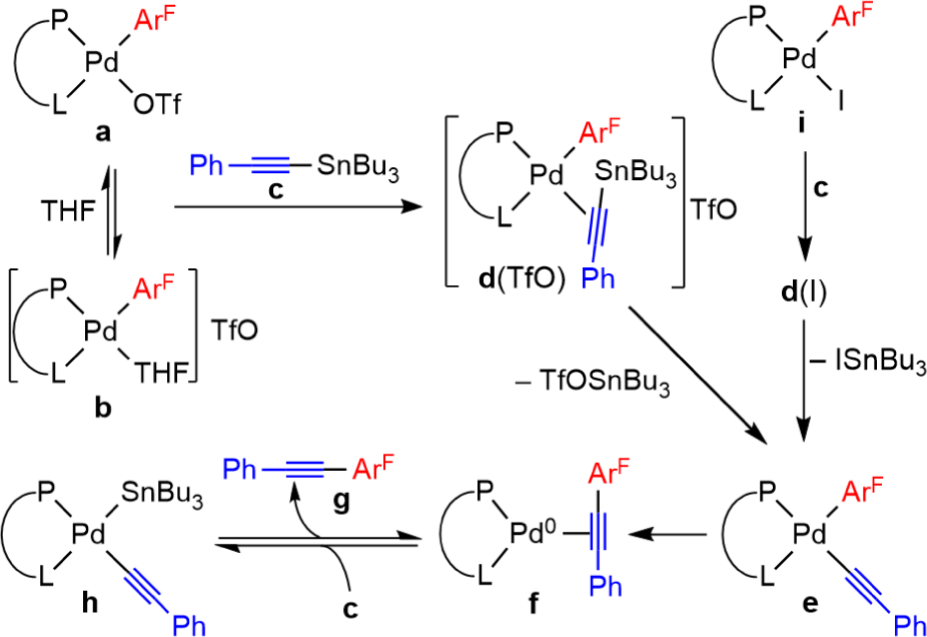
Reaction Sequence Monitored for the Reactions of [Pd(Ar^F^)X(P–L)] [X = OTf (a); X = I (i)] with an Excess of PhC≡CSnBu_3_ (c), Pd:Sn = 1:20, in THF, in the Absence of Ar^F^–I

In this section, Ar^F^ stands for C_6_F_3_Cl_2_-3,5. With the previous information, it looked still possible that the use of chelating PN ligands might prevent the formation of Pd traps such as *trans*-[Pd(Alk)(Ar^F^)L_2_]^9^ and derive cycle **I** to a different coupling pathway, that is, Shirakawa and Hiyama cycle **II**. For this reason, we recently studied the behavior of some chelating ligands in the context of Stille Ar^F^–Alk couplings intentionally frustrated by the absence of the Ar^F^–I oxidant (Scheme 2).^10^

**Figure 1:**
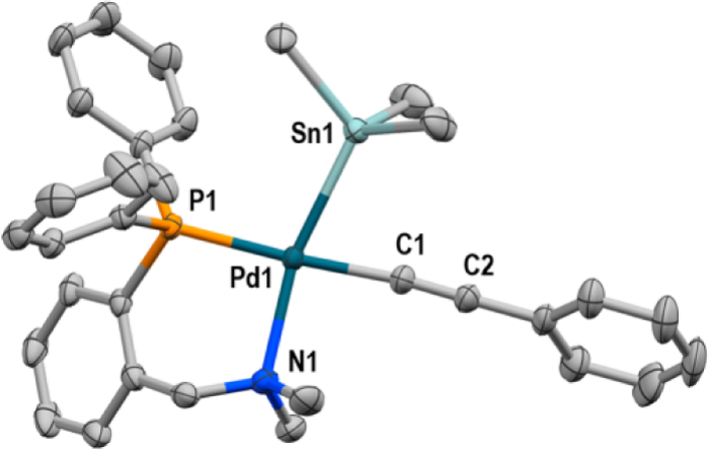
X-ray structure of **2**. H atoms and Bu groups are omitted for clarity. Relevant distances (Å) and angles (°): Pd1–Sn1 = 2.5569(3), Pd1–P1 = 2.2659(8), Pd1–N1 = 2.291(3), and Pd1–C1 = 1.993(3); C1–C2 = 1.210(5); C1–Pd1–Sn1 = 74.97(10), P1–Pd1–Sn1 = 101.75(2).

In the presence of the alynylstannane, the palladium(II) complexes [Pd(Ar^F^)X(P–L)] (X = I or OTf; P–L = dppe, and *ortho*-C_6_H_4_(PPh_2_)(CH_2_–NMe_2_)) follow the classic transmetalation + coupling evolution that reproduces part of cycle **I** and eventually leads to **2** (labeled as type **h** in Scheme 2) which is the product of the first step of the proposed cycle **II**. Fortunately, we have been able to prepare cleanly complex [Pd(Alk)(SnBu_3_)(PN)] (**2**) according to eq 1. The X-ray diffraction structure (Figure 1) confirmed the isomer suggested by the ^31^P NMR data.^11^
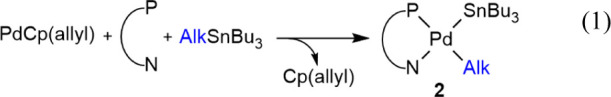
1

**Scheme 3:**
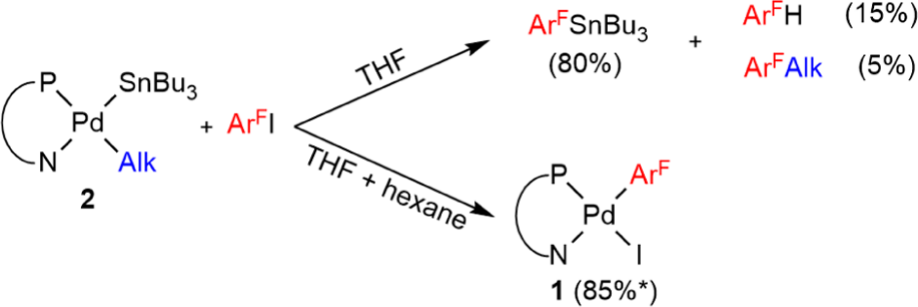
^19^F Containing Products in the Stoichiometric Reactions of 2 + Ar^F^–I at 25 °C Isolated yield.

With complex **2** in hand, we could test the dark point in cycle **II**: how does compound **2** behave in the presence of Ar^F^I? The stoichiometric reaction **2** + Ar^F^–I in neat THF (the usual solvent in the catalysis of Shirakawa and Hiyama and in the work of Crociani)^4,7^ afforded, at completion (2 days at 25 °C), 80% Ar^F^SnBu_3_ and 15% hydrolysis product Ar^F^H but only 5% of Ar^F^–Alk (Scheme 3 and Figure S1). In addition, the nonfluorinated complex [Pd(Alk)I(PN)] (**3**) was observed by ^31^P NMR. This result does not support the second step of cycle **II**, at least as a simple process.

**Scheme 4:**
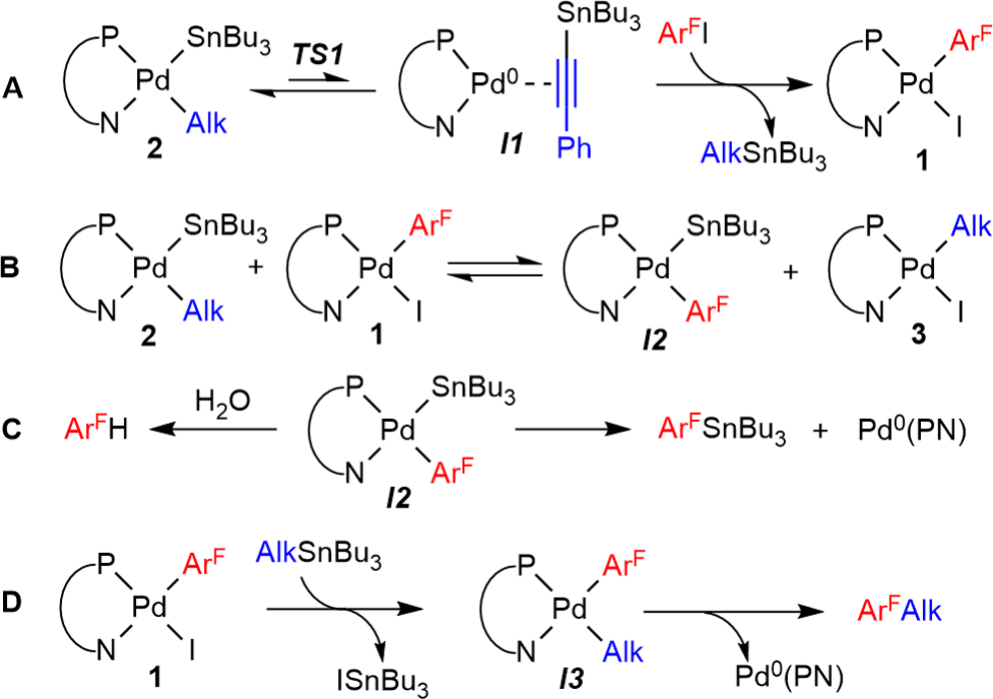
Proposed Reaction Pathways to Explain the Competitive Formation of Ar^F^–SnBu_3_, Ar^F^–H, and Ar^F^–Alk

Using THF/hexane = 2/5 (v/v) as the solvent, the result was strikingly different: [Pd(Ar^F^)I(PN)] (**1**), which is only sparingly soluble in this mixture, was isolated in high yield (≈85%), proving that **2** can be transformed into **1** via reductive elimination of **2** to Alk–SnBu_3_ + Pd^0^(PN), followed by oxidative addition of Ar^F^–I to Pd^0^(PN). This is an obvious pathway for compound **2** to re-enter cycle **I** in the form of **1**. Small amounts of Ar^F^–SnBu_3_ and Ar^F^–H were also found in the filtrate. Furthermore, we confirmed that the stoichiometric reaction of **1** + **2** in THF at room temperature leads quickly to Ar^F^–SnBu_3_ and traces of Ar^F^–H. This explains the abundant formation of Ar^F^–SnBu_3_ in THF. The conclusion is that two reactivity patterns from **2** + Ar^F^–I coexist, one that produces **1** and can bring the reaction to cycle **I**, yielding Ar^F^–Alk, and another one, so far unknown, which yields Ar^F^SnBu_3_, a product absent from the proposed cycle **II**. In the studied conditions, this second pathway is much faster than cycle **I**. Taking into account the reactivity mentioned above and the species observed in our previous study in ref (10) (Scheme 2), the reactivity model shown in Scheme 4 can be proposed.

**Figure 2:**
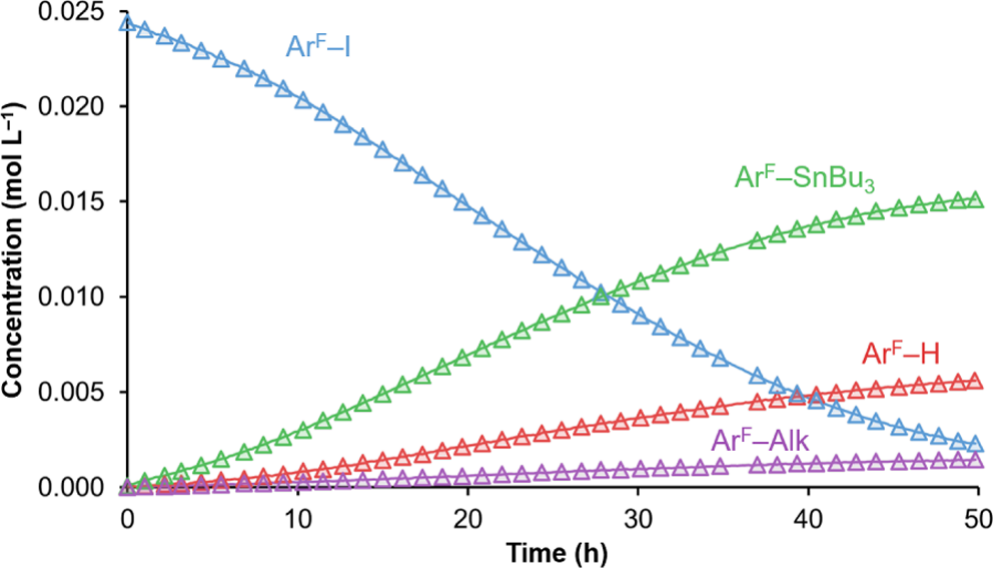
Concentration vs time ^19^F NMR monitoring data (triangles) and COPASI fitting (continuous lines) of the F-containing species in the reaction of **2** with Ar^F^–I in THF at 10 °C.

The reactions in Scheme 4 line **A** account for the formation of **1** from **2** and Ar^F^–I. In THF/hexane, complex **1** is only scarcely soluble and precipitates. However, in THF, the coexistence in a solution of **1** and **2** leads to the formation of Ar^F^–SnBu_3_ and Ar^F^–H by the sequence of lines **B**/**C** (Scheme 4).^12^ Kinetic competition of hydrolysis and Sn–C reductive elimination on ***I2*** yields Ar^F^–H and abundant Ar^F^–SnBu_3_. The fact that solutions of **1** or Ar^F^–I in wet THF are perfectly stable for days at 50 °C (temperature of catalytic conditions) confirms that the formation of Ar^F^–H requires also the presence of **2** and the formation of ***I2*** (reaction in Scheme 4, line **B**). The scarce solubility of **1** in THF/hexane reduces its presence to a very small concentration in this mixture and, consistently, limits the formation of Ar^F^–SnBu_3_ and Ar^F^–H to a very small percentage. Finally, the direct sequence of line **A** followed by line **D** completes cycle **I** and produces a small percentage of Ar^F^–Alk in arduous competition with the faster destructive competence of processes **B** and **C**. The competitive formation of the fluorinated compounds Ar^F^–SnBu_3_, Ar^F^–H, and Ar^F^–Alk in THF was ^19^F NMR monitored at 10 °C and fitted using the COPASI software (Figure 2).^13^

**Figure 3:**
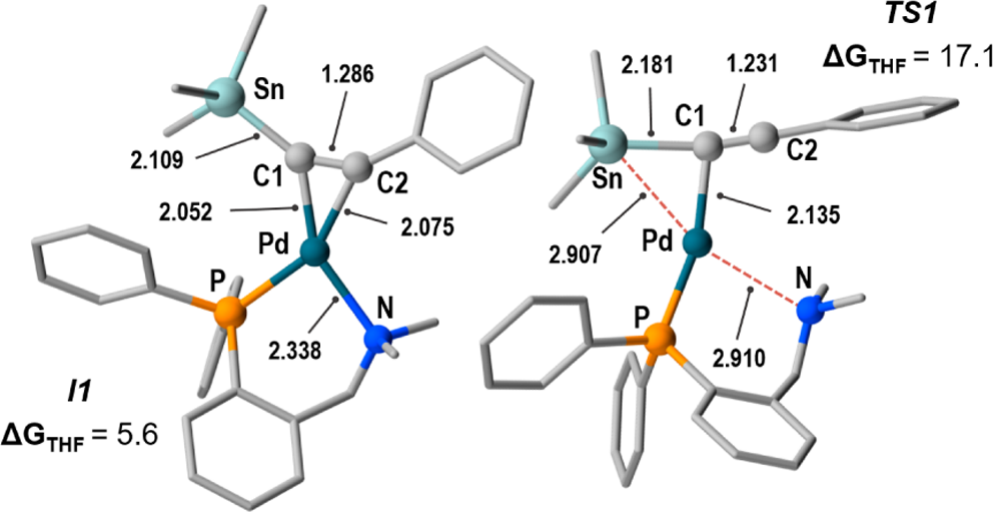
Optimized structures of ***I1***_***Me***_ (left) and ***TS1***_***Me***_ (right), using SnMe_3_ instead of SnBu_3_. Selected distances are given in Å. Δ*G*_THF_ values relative to **2_Me_** (with SnMe_3_) are given in kcal mol^–1^.

Being ***I1*** unobservable because of its minute concentration, experimental parameters for the **2** ⇋ ***I1*** equilibrium cannot be obtained and reasonable Δ*G*_0_ and Δ*G*^‡^ values from density functional theory (DFT) calculations in THF (Figure 3) were used for the COPASI fitting (full details of the DFT work on the model of Scheme 4, including optimized structures of ***I2***_***Me***_ and ***TS2***_***Me***_ in Figure S5, are given in the Supporting Information).^14^

The main curves of Figure 2 show clearly an autocatalytic effect. Note that the initial concentration of **1** in this experiment is zero until a small amount is formed following process **A** in Scheme 4. The coexistence of **1** and **2** opens autocatalytic reactivity of pathway **B** + **C**, which accelerates and becomes eventually dominant.

In conclusion, our stoichiometric study reveals the existence of an ignored pathway starting with **2** and producing efficiently Ar^F^–SnBu_3_ instead of Ar^F^–Alk. Under the specific conditions of this stoichiometric study (Alk–SnBu_3_:Ar^F^–I:Pd = 0:1:1 at 10 °C), pathway **A** + **D** of Scheme 4 which reproduces the reaction sequence of the classic Stille cycle **I**, is comparatively slow, whereas the sequence **B** + **C**, requiring the previous coexistence of **1** and **2** (achieved via **A**), is highly competitive but produces Ar^F^–SnBu_3_ and Ar^F^–H, not Ar^F^–Alk. Interestingly, in the stoichiometric reaction at a higher temperature (50 °C, the temperature used by Shirakawa and Hiyama in the catalytic experiments), the percentage of the Ar^F^–Alk (13%) and Ar^F^–H (22%) increases moderately in the detriment of Ar^F^–SnBu_3_ (65%) (Figure S2). The Ar^F^–Alk versus undesired products’ ratio increases almost 3 times from 0.052 at 25 °C to 0.149 at 50 °C, supporting higher rate acceleration with the temperature of the desired pathway **A** + **D** (Stille reaction). It seems that this tendency with temperature might explain the high coupling percentages of Ar–Alk products (yields in the order 80–90%) obtained for conventional aryls by Shirakawa and Hiyama in catalytic conditions (50 °C), but we will soon discuss that it is not that simple. Shirakawa and Hiyama did not report the existence and nature of the other products formed that account for the 100% conversions, but it is reasonable to think that Ar–SnBu_3_ products were formed, as supported by our results in the next section.

### Section B: Catalytic Studies for Ar^F^–C≡C–R Stille Coupling

**Scheme 5:**
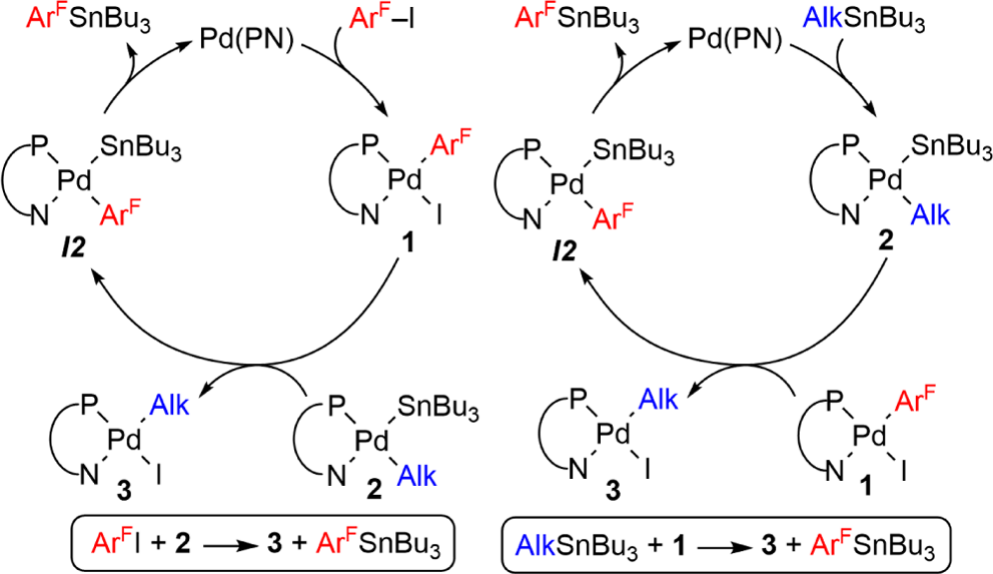
Two Cycles Forming Ar^F^–SnBu_3_ and **3** by Autocatalytic Recycling of Pd^0^(PN)

The catalytic conditions differ from the stoichiometric experiment in that the reagents AlkSnBu_3_ and Ar^F^I are in large excess relative to the Pd catalyst (e.g., 100:100:10). The Pd^0^(PN) molecules formed in the initial coupling (whether from **1** or from **2** as a catalyst) have to be recycled. The peculiarity of this reaction is that both reagents are able to produce the required oxidative addition. This gives rise to two competitive recycling processes in the second and subsequent turnovers: reoxidation with Ar^F^–I follows the cycle on the left in Scheme 5 to create **1**, whereas recycling of Pd^0^(PN) via oxidative addition with Alk–SnBu_3_ (Scheme 5, right) yields **2**. This produces the coexistence of **1** and **2** and consequently opens pathway **B** + **C** in Scheme 4 to the undesired formation of Ar^F^SnBu_3_.

**Scheme 6:**
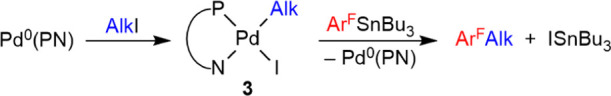
Alternative *transposed* Stille Cycle Based on the Formation of 3 in Scheme 5

At first sight, the two cycles in Scheme 5 look self-destructive since each turnover recovers one Pd in the form of **1** or **2**, producing one Ar^F^–SnBu_3_ molecule, whereas one Pd is apparently lost in the form of the out-of-cycle complex **3**. However, complex **3** can be recovered as Pd^0^(PN) in a sequence that would be part of an alternative cycle **I** starting with Alk–I and Ar^F^–SnBu_3_ instead of Ar^F^–I and Alk–SnBu_3_ (Scheme 6). We call this alternative the *transposed* catalysis and will come back to it later on. The cycles in Scheme 5 are crucial to understand the behavior of this peculiar catalytic system involving alkynylstannanes because *(i)* the existence of both cycles, re-entering the catalyst via Pd^0^(PN) oxidative addition with Alk–SnBu_3_ or with Ar^F^–I explains the mechanism of **2** ↔ **1** conversion along the catalysis; *(ii)* the coexistence of **1** and **2** activates pathway **B** + **C** that causes the formation of **3** and undesired Ar^F^–SnBu_3_; *(iii)* at the same time, the formation of **3** opens an alternative *transposed* Stille cycle that makes the undesired Ar^F^–SnBu_3_ a useful reagent to produce Ar^F^–Alk (Scheme 6). Consequently, it is a matter of finding how to improve the step rates leading to the desired evolution to Ar^F^–Alk via **1** and **3**(15) or reduce the rates leading to Ar^F^–SnBu_3_ from **2**.

The results of our first set of catalytic experiments to produce Ph-C≡C-(C_6_F_3_Cl_2_-3,5) are summarized in Table 1. The results in the absence of additives, using 10% of **2** or **1** as catalysts (entries 1 and 2), were promising: working at 50 °C, yields of Ar^F^–Alk in the range of 50–58% were achieved. The rest was undesired Ar^F^–H and Ar^F^–SnBu_3_. The yields required improvement, but the data were mechanistically meaningful. It is striking that, with complex **1** being an *in-cycle* species of the classic Stille mechanism (according to Scheme 1) and complex **2** being a disturbing species foreign to Stille cycle **I**, the results of Stille (Ar^F^–Alk) versus undesired (Ar^F^–SnBu_3_ + Ar^F^–H) yields are not very different (50/50 with **2**, 58/42 with **1**). This is due to the **2** ↔ **1** ↔ **3** conversion that, after a way, creates similar catalyst conditions.

**Table 1:**
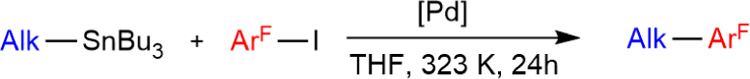
Catalytic Results of the Ar^F^–Alk Coupling with Different Catalysts and Additives^a^

entry	catalyst	additives (mol %)	Ar^F^Alk	Ar^F^SnBu_3_	Ar^F^H
1	**2** (10%)		50	28	22
2	**1** (10%)		58	33	9
3	**2** (10%)	1% AsPh_3_	85	6	9
4	**1** (10%)	1% AsPh_3_	88	2	10
5	**1** (10%)	10% AsPh_3_,	79		1
6	**4** (10%)		<1		5
7	**1** (10%)	100% LiCl	51	18	31
8	**2** (10%)	1% AsPh_3_, 100% LiCl	90		10
9	**1** (10%)	1% AsPh_3_, 100% LiCl	**>99**^b^		
10	**1** (2%)	0.2% AsPh_3_,	39	3	13
11	**1** (2%)	0.2% AsPh_3_, 100% LiCl	**96**		4
12	**1** (10%)	1% AsPh_3_, 110% LiCl	**98**^c^	2	0

a ^19^F NMR yields of each product.

b Analogous results are obtained for the reaction at 40 °C after 48 h (98%) or replacing LiCl with CsF.

c Reaction with 4-FC_6_H_4_I. 110 mol % of Alk–SnBu_3_ and LiCl are used because the first turnover from **1** can form up to 10% of PhC≡C–C_6_F_3_Cl_2_-3,5 instead of PhC≡C–C_6_H_4_F-4.

The improved competitivity of the Stille cycle observed in entries 1 and 2 (estimated by the yield in Ar^F^–Alk vs Ar^F^–SnBu_3_ + Ar^F^–H) is clearly way larger than expected from the temperature effect observed in the stoichiometric studies: 5% Ar^F^–Alk at 25 °C and only 13% at 50 °C in the stoichiometric reaction, versus 50–58% Ar^F^–Alk at 50 °C in catalysis. In the stoichiometric reaction **2** + Ar^F^–I, the evolution to produce Ar^F^SnBu_3_ through reactions **B** and **C** (Scheme 4) is, according to the results, largely dominant over the pathways **A** + **D** or **3** + Ar^F^SnBu_3_ (Scheme 6).^15^ In contrast, in catalytic conditions, the high concentrations of Ar^F^I and AlkSnBu_3_, the cycles in Scheme 5 and the Pd^0^(PN) recycling, get into play, substantially improving the competitivity of cycle I. Although **2** dominates initially in entry 1 and **1** does the same in entry 2, when the **2** ↔ **1** ↔ **3** conversions via Scheme 5 adjust their concentrations, the two catalyses come to similar success, casually close to 50% in the catalytic conditions used.

### First Catalytic Improvement

In spite of the remarkably better Ar^F^–Alk formation found in catalytic conditions, the percentage of reaction (via the Stille cycle with **1** or **3**) with a fluorinated aryl such as C_6_F_3_Cl_2_-3,5 is unsatisfactory. Fortunately, in any coupling catalysis, the initial catalyst is converted to Pd^0^ in the first turnover. If we could modify the Pd^0^(PN) ephemeral intermediate to the one that could be oxidized by Ar^F^–I but not by Alk–SnBu_3_, the undesired cycle in Scheme 5 (right), producing undesired Ar^F^–SnBu_3_, should disappear and, using **1** as a catalyst, the evolution after the first turnover should be derived to the desired cycle **I**. Similarly, starting with catalyst **2**, all of it would be converted to **1** after the first turnover and be ready to follow Stille cycle **I**.

For the sake of simplicity, we have so far represented the Pd^0^ species produced upon coupling as Pd^0^(PN), but it is unrealistic that this unsaturated molecule can survive without immediately capturing potential coordinating L ligands in solution (e.g., L = THF, OH_2_, or triple bonds of the different alkynyl-containing molecules) to give Pd^0^(PN)L_*n*_. In order to improve the catalytic results, we should simply find some appropriate coordinating L molecule as an additive that facilitates the oxidative addition by Ar^F^–I of the corresponding Pd^0^(PN)L_*n*_ as much as possible. After some unsuccessful trials, we were glad to see that the addition of AsPh_3_ in a largely substoichiometric proportion relative to the Pd catalyst (AsPh_3_:Pd = 1:10) is enough to quite efficiently quench the formation of complex **2**, producing a clear increase in the percentage of Ar^F^–Alk (>85%, Table 1, entries 3 and 4). The catalytic results confirm that the presence of substoichiometric AsPh_3_ makes the choice of catalyst **1** or **2** (entries 3 and 4) almost indifferent because if catalyst **2** is used, it only exists during the initial turnover.

This terrific effect supports that AsPh_3_ coordinates with Pd^0^(PN) in preference to the other potential ligands in solution to give Pd^0^(PN)(AsPh_3_), perhaps in equilibrium with Pd^0^(PN)(AsPh_3_)_2_. The former, more electron-rich than Pd^0^(PN) (hence more easily oxidizable) and less hindered for the approximation of the Ar^F^–I bond to the Pd atom than Pd^0^(PN)(AsPh_3_)_2_, is likely to be the most reactive one. Additionally, the presence of AsPh_3_ is able to prevent the formation of ***I1*** (Scheme 4), and hence of **2**, hampering the kinetic competition of Alk–SnBu_3_ for oxidative addition. The computed equilibrium constant for L displacement of η^2^-alkynylstannane by AsPh_3_ in Pd^0^(PN)L is *ca*. *K*_eq_ ≈ 30 at 25 °C, which supports the higher stability of Pd^0^(PN)(AsPh_3_) compared to ***I1***. On the other hand, the formation of **1** by the oxidative addition of Ar^F^–I to Pd^0^(PN) is *ca*. 35 kcal mol^–1^ more favorable than the formation of **2** by the oxidative addition of Alk–SnBu_3_ to Pd^0^(PN). Although AsPh_3_ modifies the Pd^0^(PN) species to Pd^0^(PN)(AsPh_3_), it is not consumed in the catalytic synthesis, and its role is catalytic.

Note that AsPh_3_:Pd = 1:10 is largely substoichiometric relative to the total concentration of Pd but, at the same time, largely overstoichiometric relative to the nonobservable concentration of Pd^0^(PN) just released during reductive elimination. In contrast, the addition of stoichiometric AsPh_3_ (Table 1, entry 5) is less efficient, reflecting the usual coupling retardation that almost any added ligand produces at the transmetalation step of Stille reactions.^16^ For AsPh_3_, this effect is moderate because it is not a strong ligand for Pd^II^, but entry 5 warns that the addition of AsPh_3_ should be substoichiometric (the proportion has not been optimized).

### Second Catalytic Improvement

**Figure 4:**
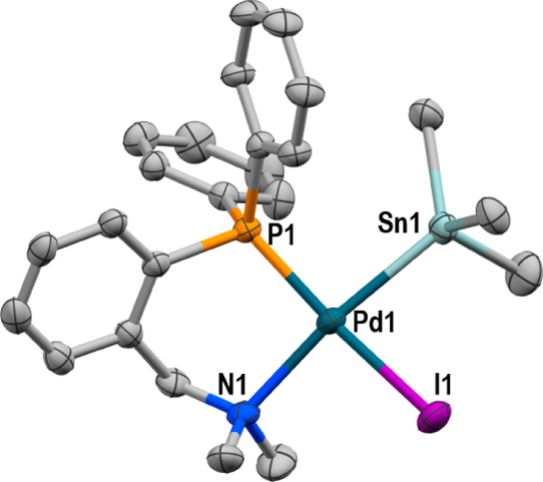
X-ray structure of **4**. Hydrogen atoms and Bu groups are omitted for clarity. Relevant distances (Å) and angles (°): Pd1–Sn1 = 2.5884(6), Pd1–N1 = 2.313(5), and I1–Pd1–Sn1 = 83.94(2).

In the catalytic reactions discussed so far, the experimental observations were mainly based on the very informative ^19^F NMR spectra. Analysis of the ^31^P NMR spectra of solutions after catalysis identified a signal with tin satellites corresponding to [PdI(SnBu_3_)(PN)] (**4**). This compound was independently synthesized for unambiguous identification by X-ray diffraction (eq 2 and Figure 4). Complex **4** is the product of the oxidative addition of I–SnBu_3_ (the byproduct of the Stille transmetalation) to Pd^0^(PN), and it turns out to be an irreversible Pd trap that precludes its re-entrance into the catalytic cycle. In fact, it was tested as a possible Pd precatalyst (Table 1, entry 6) and produced only negligible conversion.
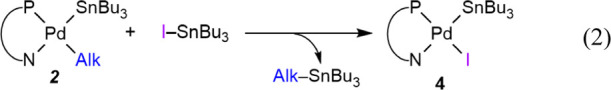
2

The accumulative formation of **4** along the catalysis can eventually reduce the active catalyst concentration to inefficient figures. LiCl (stoichiometric relative to the reactants) was added in order to transform I–SnBu_3_ into Cl–SnBu_3_, less able to oxidize Pd^0^(PN).^17^ This additive showed only moderate efficiency when alone (entry 7 vs entries 1 and 2), but in combination with 1% AsPh_3_, it brought the Ar^F^–Alk yield to 90% in the reaction with precatalyst **2** (entry 8) and to quantitative yield in the reaction with catalyst **1** (entry 9). The addition of LiCl (CsF has a similar effect) becomes more relevant for a lower percentage of the catalyst: with 2% **1** + 0.2% AsPh_3_, the catalysis only reaches 39% yield of Ar^F^–Alk before the catalytic activity expires, while the combination 2% **1** + 0.2% AsPh_3_ + stoichiometric LiCl increases this yield to 96%. Finally, entry 12 in Table 1 shows that the use of the two additives is also efficient for more conventional (less fluorinated) aryls, such as C_6_H_4_F-4, which yields 98% of 4-FC_6_H_4_–Alk and (as suspected for unreported data in the Shirakawa and Hiyama results) 2% of 4-FC_6_H_4_–SnBu_3_. This result suggests that the coupling yields reported by Shirakawa and Hiyama can also be substantially improved by the use of these additives.

### Failed or Partially Frustrated Attempts of Catalysis Improvement

In addition to the results in Table 1, we tested the reaction in entry 9 using Ar^F^–Cl instead of Ar^F^–I. It was unsuccessful, even at reflux in THF, due to its higher barrier for oxidative addition. Also, [Pd^0^(PN)(η^2^-dmfu)] (X-ray structure in Figure S7), which worked well with conventional aryls as reported by Crociani,^7^ proved inactive for Ar^F^–I + Alk–SnBu_3_ catalysis because, in contrast with Ar–I, Ar^F^–I does not undergo oxidative addition.

A very disappointing result was that when the best catalytic conditions for C_6_F_3_Cl_2_–I were applied to other fluorinated aryl iodides, significantly higher difficulty was found, depending on their fluorination (Table 2, entry 1). The almost quantitative results with 4-F-C_6_H_4_–I (entry 2) show that this aryl practically displays the behavior not far from what we call “conventional” aryls, but for other fluorinated aryls, a very significant drop of yield is observed. The effect is particularly high when one or (more markedly) the two positions *ortho* to the *ipso*-C atom are fluorinated (entries 3 and 4).

**Table 2:** Catalytic Ar^F^–Alk Results of Ar^F^–I + PhC≡C–SnBu_3_ Coupling Catalyzed by Complex 1, with Different Fluorinated Aryl Groups^a^

entry	Ar^F^I	catalyst	Ar^F^I	Ar^F^Alk	Ar^F^H
**1**^b^	3,5-C_6_F_3_Cl_2_I	**1** (10%)	0	>99	0
2^c^	4-FC_6_H_4_I	**1** (10%)	0	98^d^	0
3	2-FC_6_H_4_I	**1** (10%)	11	82	7
4	2,6-F_2_C_6_H_3_I	**1** (10%)	70	30	0
5^e^	2,6-F_2_C_6_H_3_I	**1** (10%)	10	70	10^f^
6	C_6_F_5_I	**1** (10%)	4	70	26
7^g^	C_6_F_5_I	**1** (2%)	3	70	27

a Reaction conditions as in entry 9 of Table 1.

b Entry 9 of Table 1.

c Entry 12 of Table 1.

d 2% Ar^F^SnBu_3_.

e 12 h at 100 °C.

f Plus others (10%).

g 24 h, 90 °C, 1,4-dioxane.

The specific problem of highly fluorinated aryls is that, due to the high group electronegativity, their nucleophilic reagents (e.g., C_6_F_5_–SnBu_3_) are weaker than conventional aryls. On the other hand, the corresponding electrophilic reagents producing the oxidative addition (e.g., C_6_F_5_–I) are also less reactive than their nonfluorinated congeners.^18,19^ For electron-rich Pd^0^ complexes, even C_6_F_5_–I reacts sufficiently well, but the poor electron density of Pd^0^(PN) worsens the problem. The fact that increasing the reaction temperature to 90–100 °C (entries 5 and 7) very significantly improves the conversion indicates that more fluorinated reagents are finding higher energetic barriers in the oxidative addition or in other catalytic steps. Yet, at the limit of fluorination (C_6_F_5_, entry 7), an acceptable 70% yield of the coupling product C_6_F_5_–C≡C–Ph can be achieved with just 2% of catalyst **1**, although at 90 °C and suffering 27% of hydrolysis.

### More Practical Alternative?: The “Transposed” Catalysis

The solutions so far applied to improve the catalysis are based on trying to make the oxidative addition reaction Ar^F^I + Pd^0^(PN) more efficient than Alk–SnBu_3_ + Pd^0^(PN). Obviously, in the mechanistic study in Section A, we could not alter the combination of reagents used by Shirakawa and Hiyama, Ar–I + Alk–SnBu_3_, but for catalysis, this restriction does not hold. Moreover, in Scheme 5, we have found that the initial conversion **2** ↔ **1** and the coexistence of both complexes in solution lead to the formation of [Pd(Alk)I(PN)] (**3**) and Ar^F^–SnBu_3_, also able to follow a Stille cycle. Both Stille cycles, C_6_F_3_Cl_2_–I + AlkSnBu_3_ (cycle **I** in Scheme 1) and its transposed version (Scheme 6) are able to produce the desired Ar^F^–Alk product. It is probably hopeless to approach a mechanistic study on so many products, steps, and barriers. From a practical view, it is more reasonable simply to check the catalytic efficiency when the groups (Ar^F^ and Alk) *transpose* their roles.

A recently published DFT study at the ZORA-BLYP/TZ2P level has shown that the activation energy for the oxidative addition of C(sp^*n*^)–X bonds (*n* = 1–3; X = H, Me, Cl) to Pd^0^L_*n*_ is lower for a lower number of C substituents. Hence, it is the lowest for alkynyl groups (*n* = 1).^20^ Assuming that this will hold for X = SnBu_3_, the oxidative addition of the Alk–SnBu_3_ bond (*n* = 1) should be favored by this effect compared to Ar^F^–I (*n* = 2). Moreover, the entropically disfavored associative step of oxidative addition should be better compensated by the stronger coordination to Pd^0^ of the C≡C triple bond compared to Ar^F^. We suggest that these two circumstances concur to facilitate the oxidative addition of Alk–SnBu_3_ and create the alkynylstannane problem when combining Ar^F^–I (only weak π-donors) and alkynylstannanes (stronger π-donors), which is not observed for other stannane reagents. According to this analysis, the lower oxidative addition barriers in a *transposed* Stille cycle, with Csp atoms versus Csp^2^ and the easier coordination to Pd^0^(PN), should facilitate the Alk–I oxidative addition, easing the direct formation of [Pd(Alk)I(PN)] (**3**). A subsequent transmetalation reaction with Ar^F^–SnBu_3_ should continue the transposed Stille cycle.

Complex **3** was prepared from **2** taking advantage of the easy reductive elimination of AlkSnBu_3_ to ***I1*** (eq 3) to be used as a catalyst. Complex [Pd(Alk)Cl(PN)] (**5**), used also in Table 3, can be prepared similarly using Cl–Alk instead of I–Alk (see full characterization and synthetic details in the Supporting Information, including the X-ray structure of **5** in Figure S6).
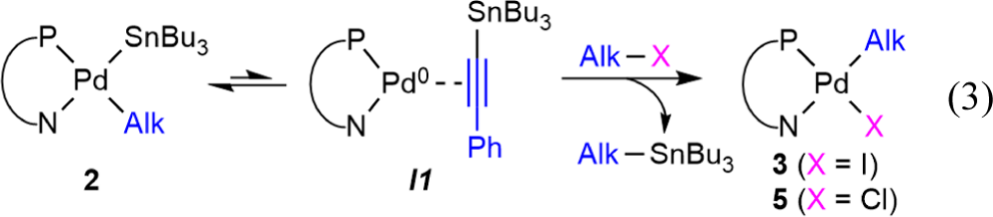
3

**Table 3:**
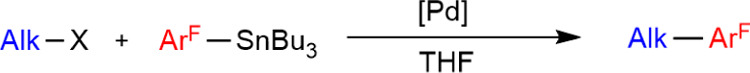
Catalytic Results of the Transposed Ar^F^–Alk Coupling Using Alk–X (X = Cl, I), Ar^F^–SnBu_3_, and Catalysts **2**, **3**, or **5**^a^

entry	Pd catalyst	additives	Ar^F^–Alk	Ar^F^–Sn	Ar^F^–H
1	**3** (10%) *		81	18	1
2	**3** (10%) *^,^^b^^,^^c^	AsPh_3_, LiCl	>99		
3	**3** (10%) *^,^^c^	LiCl	>99		
4	**3** (2%) *^,^^b^	AsPh_3_	68	29	3
5	**3** (2%) *^,^^c^	LiCl	98		2
6	**5** (2%) **		31	65	4
7	**5** (2%) **^,^^b^	AsPh_3_	97		3
8	**2** (10%) **^,^^d^		21	51	28
9	**2** (10%)**^,^^b^	AsPh_3_	89	6	5
10	**2** (2%) **^,^^b^	AsPh_3_	82	12	6

a Ar^F^ = C_6_F_3_Cl_2_-3,5.

b Substoichiometric AsPh_3_ (10 mol % with respect to the Pd catalyst).

c Stoichiometric LiCl (100 mol %).

d Only 49% conversion. *Alk–I is used as a reactant and the reaction is left for 24 h at 50 °C. **Alk–Cl is used as a reactant and the reaction is left for 36 h at 70 °C.

**Scheme 7:**
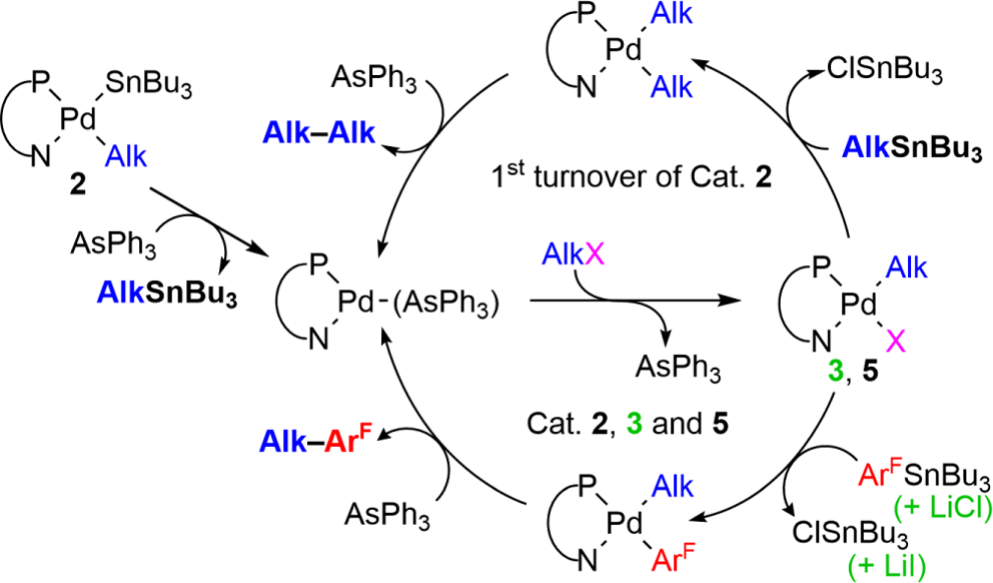
In Situ Formation of Catalysts **3** and **5** from 2 and Routes for the Formation of the Alk–Alk Byproduct and the Heterocoupling Product Alk–Ar^F^ Li salts used only for catalyst **3**. Ar^F^ = C_6_F_3_Cl_2_.

The results of several transposed catalyses with **2**, **3**, or **5** as catalysts are presented in Table 3, and the catalytic cycles applied in each case are illustrated in Scheme 7. The analysis of the results reveals a bonus in the transposed protocol and provides a finer appreciation of the role of each additive.

Scheme 7 is represented assuming the formation of Pd^0^(PN)(AsPh_3_) after the first turnover in the presence of substoichiometric AsPh_3_. This is not the case for several entries (for instance, with 10 mol % of catalyst **3**, entries 1 and 2 show that the use of stoichiometric LiCl, either with or without substoichiometric AsPh_3_, increases the yield of C_6_F_3_Cl_2_–Alk from 81% to quantitative). With 2% catalyst and 0.2% AsPh_3_ (entries 4 and 5), only 68% conversion to Ar^F^–Alk is achieved, and stoichiometric LiCl is still needed to keep the catalyst active, hampering the formation of [PdI(SnBu_3_)(PN)] (**4**). Practically, a quantitative yield (98%) is obtained with 2% of **3** in 24 h at 50 °C. The addition of AsPh_3_ seems almost unnecessary or little effective in reactions with Alk–I (entries 1–5) but is required (as well as a higher temperature) for the otherwise slow oxidative addition with Alk–Cl (entries 6–10). This is clearly observed when comparing entries 6 and 7, with **5** as a catalyst.

Catalyst **5**, with X = Cl, was prepared because should the oxidative addition be active with Alk–Cl, the transmetalation byproduct would be the nondisturbing Cl–SnBu_3_ instead of I–SnBu_3_. Then, we could spare the stoichiometric LiCl, making the reaction significantly more atom-economic. As the oxidative addition of chlorides has a higher barrier than iodides, Alk–Cl, with [Pd(Alk)Cl(PN)] (**5**) as the catalyst (entry 6), notably slowed the catalytic rate, but the addition of substoichiometric AsPh_3_ and increase of the reaction temperature had again a clear accelerating effect (entry 7), providing 97% yield of Ar^F^–Alk in 36 h at 70 °C with only 2% **5** and 0.2% AsPh_3_.^21^ Although the increase in temperature causes a slight increase of hydrolysis, this method is probably the cleanest and more practical catalytic protocol.

With **2** as the catalyst (Table 2, entries 8–10), the initial turnover on **2** stoichiometrically produced AlkSnBu_3_ (up to 10%), which reacted with the oxidative addition product [Pd(Alk)Cl(PN)] (**5**) to give [Pd(Alk)_2_(PN)] and then Alk–Alk. The formation of the latter was confirmed by GC–MS (Scheme 7, cycle pathway above). To compensate for this loss of Alk–Cl reagent, its proportion was increased in the percentage of catalyst being used (e.g., 110 mol % of Alk–Cl if 10% of the catalyst is used). Again, the catalysis with **2** was comparatively slow in the absence of AsPh_3_ (entry 8, only 49% conversion and 21% yield), but it worked reasonably well with 10 mol % of **2** and 1 mol % of AsPh_3_ (up to 89% yield) or with 2 mol % of **2** and 0.2 mol % of AsPh_3_ (82% yield). This proves efficient in situ formation, in catalytic conditions, of Pd^0^(PN)(AsPh_3_)_*n*_ and therefrom **5** (Scheme 7, upper cycle).

Table 4 shows the catalytic results of the transposed catalysis for the challenging C_6_F_5_ aryl. It produces C_6_F_5_–Alk in high yield (87%) and with little hydrolysis (Table 4, entry 2) using Alk–Cl, 10 mol % of **5**, and 1 mol % of AsPh_3_. The amount of the catalyst can be reduced to 2% operating at 90 °C, with a significant reduction of conversion.

**Table 4:**
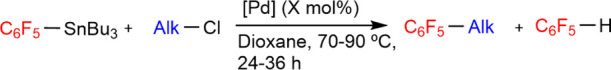
Catalytic Results of C_6_F_5_–Alk Couplings Using Alk–Cl and C_6_F_5_–SnBu_3_ Catalyzed by **5**^a^

entry	*t* (h)	*T* °C	catalyst	SM	C_6_F_5_–Alk	C_6_F_5_–H
1^b^	24	70	**5** (10%)	32	61	7
2^b^	36	70	**5** (10%)	0	87	13
3^b^	36	90	**5** (2%)	21	72	7

a SM = starting material.

b Substoichiometric AsPh_3_ (10 mol % with respect to the Pd catalyst).

Finally, since all the previous catalytic reactions use the hemilabile PN ligand, which presumably facilitates the transmetalation and reductive elimination steps by easy N dissociation,^22^ we decided to check the activity of [PdCl_2_(Ph-PEWO-F)] (**6**), bearing a phosphine-olefin ligand, also hemilabile in Pd^II^ and designed to facilitate difficult couplings.^23^ Because it makes Pd^0^ oxidation to Pd^II^ more difficult, C_6_F_5_–I was used. An in situ formed Buchwald complex with tBuXPhos, which displays a similar ability to promote challenging couplings,^24^ was also tested (Table 5).

**Table 5:** Catalytic Results of C_6_F_5_–Alk Couplings Using C_6_F_5_–I and Bu_3_Sn–C≡CPh or the Transposed Combination Using Alk–Cl, Catalyzed by [PdCl_2_(Ph-PEWO-F)] (6) or {[PdCl_2_(CH_3_CN)_2_] + tBuXPhos} (7)^a^^,^^b^

entry	R′–Sn	R–X	Cat	SM	R–R′	C_6_F_5_H
1^c^^,^^d^	PhCC–Sn	C_6_F_5_–I	**6** (5%)	5	85	6^e^
2^d^	C_6_F_5_–Sn	PhCC–Cl	**6** (5%)	30	30	40
3^c^^,^^f^	PhCC–Sn	C_6_F_5_–I	**7** (10%)	<1	86	14
4^f^	C_6_F_5_–Sn	PhCC–Cl	**7** (10%)	58	1	41

a SM = starting material.

b Reaction conditions: 24 h, 80 °C, 1,4-dioxane. R–X (1 equiv).

c Stoichiometric LiCl (100 mol %).

d R′–SnBu_3_ (1.1 equiv).

e 4% of unknown Pd(C_6_F_5_) species.

f R′–SnBu_3_ (1.2 equiv.)

The results using Alk–Cl and C_6_F_5_–SnBu_3_ (entries 2 and 4) were unexpectedly disappointing, with high hydrolysis percentages, but those using C_6_F_5_–I and Alk–SnBu_3_ (entries 1 and 3) were very satisfactory as an alternative that works well in dioxane at 80 °C only with stoichiometric LiCl.

### Stille Catalysis for the Heterocoupling of Alkynyls

The positive results in entries 9 and 10 of Table 3 and GC–MS confirmed that the formation of Alk–Alk products, indicating that the homocoupling of alkynyls (upper cycle of Scheme 7) can be promoted with our PN ligand platform, is an invitation to test the Stille catalysis for the heterocoupling of alkynyls. There are many reports for efficient and selective catalytic homo- and heterocoupling of alkynyls,^25^ but a Stille process, in case it is needed for reasons of compatibility with sensitive groups, is lacking. Table 6 collects some tests for the catalytic synthesis, as an example, of the unsymmetrical 1,3-diyne ^*t*^BuC≡C–C≡CPh using ^t^BuC≡C–I, Bu_3_Sn–C≡CPh, and **2** as precatalyst.

**Table 6:**
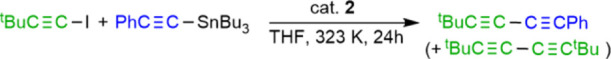
Catalytic Results of Alk–Alk′ Couplings Using ^*t*^Bu–C≡C–I and Bu_3_Sn–C≡CPh Catalyzed by **2**

entry	catalyst (%)	additives^a^	^*t*^BuC_2_–C_2_Ph	^*t*^BuC_2_–C_2_^*t*^Bu	^*t*^BuC_2_–I
1	10	AsPh_3_, LiCl	92	8	
2	10	LiCl	92	8	
3	10		91	9	
4	2	LiCl	91	9	
5	2		58	6	36

a 1 mol % AsPh_3_ and 100 mol % LiCl when specified.

The conversions are quantitative for entries 1–4, with high selectivity toward heterocoupling, over the homocoupling of the electrophile (92:8 or 91:9 by GC–MS). Since in this case, **2** only acts as a precursor of the Pd^0^ species and the oxidative addition is fast, the effect of AsPh_3_ is negligible (entry 1 vs entry 2). The effect of LiCl is unnoticed with 10 mol % of the catalyst (entry 2 vs 3) and becomes evident only with 2 mol % of the catalyst in the absence of LiCl protection (entry 5 vs 4). Needless to say, this method can be applied to the synthesis of symmetric dialkynes if using reagents with Alk = Alk′.

## Conclusions

Our study demonstrates that catalytic cycle **II** in Scheme 1, so far accepted to be a mechanistic exception operating in Stille reactions with alkynylstannanes, must be discarded as such because reacting [Pd(Alk)(SnBu_3_)(PN)] (**2**) with Ar^F^–I, it mainly produces Ar^F^–SnBu_3_ instead of Alk–Ar^F^. However, an alternative Stille cycle starting with the *transposed* products Ar^F^–SnBu_3_ + Alk–X as reagents (Scheme 6) may also form Alk–Ar^F^ in moderate to high yield depending on the exact nature of Alk–X (X = I, Cl). Remarkably, the *direct* Stille cycle **I** (Scheme 1) starting with Ar–I, Alk–SnBu_3_, and Pd^0^(PN) can be made preferred by preventing the formation of undesired Pd–Sn species using special protocols. These are absolutely necessary for fluorinated aryls: *(i)* the addition of substoichiometric percentages of AsPh_3_ (AsPh_3_: Pd = 1:10) to give Pd^0^(PN)(AsPh_3_) blocks the formation of **2**, making the direct Stille catalysis more competitive; *(ii)* the addition of stoichiometric LiCl relative to the Sn reagent (Cl/Sn = 1:1) hinders the formation of [PdI(SnBu_3_)(PN)] (**4**), a Pd trap. The use of the two additives brings the catalytic results of aryl fluorinated alkynes and, of course, those of conventional aryls, close to quantitative; *(iii)* a more radical solution to the problems in catalysis associated with the use of alkynylstannanes is to avoid its use, employing instead the *transposed* combination of reagents. Moreover, with Alk–Cl as the electrophile (e.g., Alk–Cl + Ar^F^–SnBu_3_), this method skips the need for LiCl. This procedure works great with just a minimal substoichiometric percentage of AsPh_3_ as an additive (2 mol % of the Pd catalyst and 0.2 mol % of AsPh_3_) and even without AsPh_3_ in some cases. In summary, the classic Stille catalysis rules the Ar–Alk couplings, provided that the particular idiosyncrasy of alkynylstannanes as potential oxidative addition reagents is understood and accordingly dealt with.

The mechanisms discussed in this study for Ar^F^ reagents are shared by the conventional aryls but only produce very problematic results when fluorinated aryls are involved due to the more challenging oxidative addition of Ar^F^–X electrophiles (in the direct Stille reaction) and the very low nucleophilicity of Ar^F^–SnBu_3_ nucleophiles (in the transposed Stille reaction), compared to their congeners with conventional aryls.

In this general study, the reaction temperatures and times or the minimum percentages of catalysts or additives have not been exhaustively optimized and there is space for further improvement in specific cases. The problems and solutions in this work could be applied to make other R–Alk Stille couplings feasible, as shown for the efficient synthesis of unsymmetrical 1,3-diynes. We wish to remark on the splendid catalytic effect of AsPh_3_ which, in substoichiometric amounts relative to the metal catalyst, precludes the formation of undesired intermediates in kinetically efficient concentrations. In this respect, we refer the reader to a related case (different in its intimate details) occurring in gold catalysis.^26^

## Supporting Information Available

Synthesis and full characterization of the metal complexes, NMR spectra (^1^H, ^13^C, ^19^F, ^31^P, and ^119^Sn), kinetic and microkinetic details, DFT section, and catalytic experiments (PDF)
