# Supplementary material for: Problematic ArF–Alkynyl Coupling with Fluorinated Aryls. From Partial Success with Alkynyl Stannanes to Efficient Solutions via Mechanistic Understanding of the Hidden Complexity
Source: J Am Chem Soc. 2022 Dec 21;145(1):527–36. doi: 10.1021/jacs.2c10842 (PMC9837839; doi:10.1021/jacs.2c10842)
Supplement: Supplementary file 1 — ja2c10842_si_001.pdf [file ja2c10842_si_001.pdf]

## Supporting Information

# Problematic $Ar^F$–Alkynyl Coupling with Fluorinated Aryls. From Partial Success with Alkynyl Stannanes to Efficient Solutions via Mechanistic Understanding of the Hidden Complexity

Guillermo Marcos-Ayuso,[a] Marconi N. Peñas-Defrutos,[a,b] Ana M. Gallego,[a] Max García-Melchor,[b] Jesús M. Martínez-Ilarduya,[a] and Pablo Espinet*[a]

[a] IU CINQUIMA/Química Inorgánica, Facultad de Ciencias, Universidad de Valladolid, E–47071 Valladolid, Spain

[b] School of Chemistry, CRANN and AMBER Research Centres, Trinity College Dublin, College Green, Dublin 2, Ireland

E–mail: espinet@qi.uva.es

## Table of Contents


**1. General information** .......... **3**

**2. Synthesis and characterization of the metal complexes** .......... **4**

**Synthesis of [Pd(C≡CPh)($SnBu_3$)(PN)] (2)** .......... 4

**Synthesis of [Pd(Alk)I(PN)] (3)** .......... 4

**Synthesis of [PdI($SnBu_3$)(PN)] (4)** .......... 5

**Synthesis of [Pd(Alk)Cl(PN)] (5)** .......... 5

**Alternative synthesis of [Pd($Ar^F$)I(PN)] (1)** .......... 5

**Synthesis of [$PdI_2$(PN)]** .......... 6

**Synthesis of [Pd(PN)($\eta^2$-dmfu)]** .......... 6

**3. Mechanistic support: Kinetic experiments** .......... **8**

**4. Mechanistic support: Computational Studies** .......... **11**

**5. Catalytic experiments** .......... **14**

**Reaction Conditions (Table 1 & S2)** .......... 14

**Characterization of the products** .......... 15

**Reaction Conditions (Table 2 & S3)** .......... 16

**Reaction Conditions (Table 3 & S4)** .......... 20

**Reaction Conditions (Table 4 & S5)** .......... 18

**Reaction Conditions (Table 5 & S6)** .......... 19

**6. X-ray diffraction details** .......... **21**

**7. NMR spectra: $^1H$, $^{13}C$, $^{19}F$, $^{31}P$ and $^{119}Sn$** .......... **24**

**8. References** .......... **35**

# 1. General information

All manipulations were carried out under a nitrogen or argon atmosphere using standard Schlenk, vacuum, cannula, or glovebox techniques. Tetrahydrofuran (THF), diethyl ether ($Et_2O$), $CH_2Cl_2$ and n-hexane were obtained oxygen and water free from an SPS PS–MD–5 solvent purification apparatus. $CDCl_3$ was dried by the usual procedure and distilled under argon prior to be used.[1] THF-$d_8$ was used without further purification.

The technical measurements were carried out with equipment of the LTI services or the IU CINQUIMA (both of the University of Valladolid) unless otherwise stated.

$^1$H, $^{19}$F, $^{31}$P{$^1$H}, $^{13}$C{$^1$H} and $^{119}$Sn{$^1$H} NMR spectra were recorded on a Varian 500/54 Premium Shielded instrument. Chemical shifts (in δ units, parts per million) were referenced to the residual solvent peaks ($^1$H and $^{13}$C), $CCl_3F$ ($^{19}$F), $SnMe_4$ ($^{119}$Sn) or external 85% $H_3PO_4$ ($^{31}$P), at 298 K, unless otherwise stated. In those cases, the temperature for the NMR probe was calibrated with an ethylene glycol standard (high temperature) and with a methanol standard (low temperature).[2] In the $^{19}$F spectra registered in non-deuterated solvents, a coaxial tube containing acetone-$d_6$ was used to maintain the $^2$H lock signal. Coupling constants (*J*) are given in hertz (Hz). The following abbreviations are used to describe peak patterns when appropriate: s (singlet), d (doublet), t (triplet), q (quartet), m (multiplet), and br (broad).

The elemental analyses were performed by the Elemental Analysis Unit of the University of Vigo on a Carlo Erba 1108 CHN analyzer.

Commercially available chemicals were purchased from Sigma Aldrich, Alfa Aesar, Fluorochem and Acros Organics companies and were used without further purification: 1,3,5-trichloro-2,4,6-trifluorobenzene (Fluorochem), trifluorotoluene (Fluorochem), (Chloroethynyl)benzene (Aldrich), 4-fluoroiodobenzene (Fluorochem), tributyl(phenylethynyl)tin (95%, Aldrich), KI (Acros), LiCl (Acros), CsF (Alfa Aesar), tributyltin iodide (90%, Aldrich), triphenylarsine (Fluorochem), $Pd_2(dba)_3{\cdot}CHCl_3$ (Aldrich) and tBuXPhos (Aldrich). Other products were synthesized as previously reported: [PdCp(allyl)],[3] $Ar^F$–H ($Ar^F$ = $C_6F_3Cl_2$-3,5),[4] $PPh_2(C_6H_4\text{-}CH_2NMe_2\text{-}2)$ (PN from now),[5] $Ar^F$–I,[6] $Ar^F$–$SnBu_3$,[7] $Ar^F$–C≡C–Ph,[8] [Pd($Ar^F$)I(PN)] (**1**),[9] [$PdCl_2(NCMe)_2$],[10] $Ar^{F'}$–C≡C–Ph ($Ar^{F'}$ = $C_6H_4F$-4),[11] Ph–C≡C–I,[12] Ph–C≡C–Cl,[13] and $^t$Bu–C≡C–C≡C–Ph,[14] $Ar^{F'}$–C≡C–Ph ($Ar^{F'}$ = $C_6F_5$), [15] [$PdCl_2$(Ph-PEWO-F)],[16] $Ar^{F'}$–C≡C–Ph ($Ar^{F'}$ = 2,6-$F_2C_6H_3$),[17] $Ar^{F'}$–C≡C–Ph ($Ar^{F'}$ = 2-$FC_6H_4$).[18]

## 2. Synthesis and characterization of the metal complexes

**Synthesis of [Pd(C≡CPh)(SnBu$_3$)(PN)]** (**2**)

In a glovebox, [PdCp(allyl)] (101 mg, 0.475 mmol), PhC≡CSnBu$_3$ (176 µL, 0.475 mmol) and the PN ligand (152 mg, 0.476 mmol) were added over 3 mL of dry THF. The mixture was stirred at room temperature for 5 min, and then n-hexane (5 mL) was added to afford a microcrystalline colorless solid, which was washed with cold n-hexane (2 × 3 mL) and vacuum dried. Yield: 334 mg (86 %).

Crystals valid for X-ray diffraction analysis were obtained by slow diffusion of n-hexane in a solution of the compound in THF (see molecular structure in Figure 1).

Anal. Calcd for $C_{41}H_{54}NPPdSn$: C 60.28; H, 6.66; N, 1.71. Found: C, 60.29; H, 6.65; N, 1.72.

$^1$H NMR (499.72 MHz, THF-$d_8$): δ 7.65–7.25 (m, 15H, PPh$_2$ + C≡CPh + C$_6$H$_4$), 7.12 (m, 2H, PPh$_2$), 7.02 (m, 1H, C$_6$H$_4$), 6.72 (m, 1H, C$_6$H$_4$), 3.34 (br, 2H, CH$_2$), 2.64 (br, 6H, Me), 1.50 (m, 6H, CH$_2$), 1.22 (m, 6H, CH$_2$), 0.79 (t, 9H, $^3J_{H\text{-}H}$ = 7.3 Hz), 0.68 (m, sat. 6H, $^2J_{H\text{-}Sn}$ = 41.7 Hz, SnCH$_2$).

$^{31}$P{$^1$H} NMR (202.31 MHz, THF-$d_8$): δ 28.7 (s, sat. $^2J_{P\text{–}Sn}$ = 15.8 Hz).

$^{119}$Sn{$^1$H} NMR (186.35 MHz, THF-$d_8$): δ –5.2 (d, $^2J_{P\text{–}Sn}$ = 15.8 Hz).

**Synthesis of [Pd(Alk)I(PN)] (3)**

In a glovebox, Ph–C≡C–I (13.7 mg, 0.060 mmol) was added to a solution of [Pd(C≡CPh)(SnBu$_3$)(PN)] (**2**) (49 mg, 0.060 mmol) in 2 mL of dry THF. The mixture was vigorously stirred for 15 min at room temperature, and then n-hexane (5 ml) was added to afford a microcrystalline orange solid, which was washed with cold n-hexane (2 × 2 mL) and vacuum dried. Yield: 33 mg (84 %).

Anal. Calcd for $C_{29}H_{27}INPPd$: C, 53.27; H, 4.16; N, 2.14. Found: C, 53.41; H, 3.99; N, 2.19.

$^1$H NMR (499.72 MHz, CDCl$_3$): δ 7.9–7.3 (m, m, 13H, PPh$_2$ + C$_6$H$_4$), 6.95 (m, 3H, C≡C–Ph), 6.83 (m, 1H, C$_6$H$_4$), 6.60 (m, 2H, C≡C–Ph), 3.8–2.5 (vbr, 8H, CH$_2$ + Me).

$^{31}$P{$^1$H} NMR (202.31 MHz, CDCl$_3$): δ 19.2 (s).

**Synthesis of [PdI(SnBu$_3$)(PN)] (4)**

In a glovebox, ISnBu$_3$ (19 µL, 0.060 mmol) was added to a solution of [Pd(C≡CPh)(SnBu$_3$)(PN)] (**2**) (49 mg, 0.060 mmol) in 2 mL of dry THF. The mixture was stirred at room temperature for 10 min, and then n-hexane (5 mL) was added to afford a microcrystalline yellow solid, which was washed with cold n-hexane (2 × 2 mL) and vacuum dried. Yield: 40 mg (79 %).

Crystals valid for X-ray diffraction analysis were obtained by slow diffusion of n-hexane in a solution of the compound in THF (see molecular structure in Figure 4).

Anal. Calcd for $C_{33}H_{49}INPPdSn$: C, 47.03; H, 5.86; N, 1.66. Found: C, 47.24; H, 5.98; N, 1.55.

$^{1}$H NMR (499.72 MHz, THF-*d$_8$*): δ 7.75–7.20 (m, 13H, $PPh_2$ + $C_6H_4$), 6.62 (m, 1H, $C_6H_4$), 3.3 (vbr, 2H, $CH_2$), 2.5 (vbr, 6H, Me), 1.43 (m, 6H, $CH_2$), 1.21 (m, 6H, $CH_2$), 0.95–0.70 (m, 15H, Me + $SnCH_2$).

$^{31}$P{$^{1}$H} NMR (202.31 MHz, THF-*d$_8$*): δ 33.4 (s, sat. $^{2}J_{P\text{–}Sn}$ = 53.8 Hz).

$^{119}$Sn{$^{1}$H} NMR (186.35 MHz, THF-*d$_8$*): δ 31.4 (d, $^{2}J_{P\text{–}Sn}$ = 53.8 Hz).

**Synthesis of [Pd(Alk)Cl(PN)] (5)**

In a glovebox, Ph–C≡C–Cl (73.7 mg, 0.540 mmol) was added to a solution of [Pd(C≡CPh)(SnBu$_3$)(PN)] (**2**) (147 mg, 0.180 mmol) in 5 mL of dry THF. The mixture was vigorously stirred for 15 min at room temperature, and then n-hexane (15 ml) was added to afford a yellow solid, which was washed with cold n-hexane (2 × 3 mL) and vacuum dried. Yield: 81 mg (80 %).

Anal. Calcd for $C_{29}H_{27}ClNPPd$: C, 61.94; H, 4.84; N, 2.49. Found: C, 61.68; H, 4.72; N, 2.56.

$^{1}$H NMR (499.72 MHz, $CDCl_3$): δ 7.8–7.3 (m, 13H, $PPh_2$ + $C_6H_4$), 6.95 (m, 3H, C≡C–Ph), 6.82 (m, 1H, $C_6H_4$), 6.64 (m, 2H, C≡C–Ph), 3.70–2.60 (vbr, 8H, $CH_2$ + Me).

$^{31}$P{$^{1}$H} NMR (202.31 MHz, $CDCl_3$): δ 24.4 (s).

**Alternative synthesis of [Pd(Ar$^{F}$)I(PN)] (1)**

In a glovebox, Ar$^{F}$I (19.6 mg, 0.060 mmol) was added to a solution of [Pd(C≡CPh)(SnBu$_3$)(PN)] (**2**) (49 mg, 0.060 mmol) in 2 mL of dry THF. The mixture was vigorously stirred for 2 min at room temperature, and then n-hexane (5 ml) was added to afford a microcrystalline yellow solid, which was washed with cold n-hexane (2 × 2

mL) and vacuum dried. Yield: 37 mg (82 %). $^1$H and $^{19}$F, $^{31}$P{$^1$H} NMR spectra were consistent with reported data.[9]

* Note that the presence, in the mother liquors of the reaction, of $Ar^F$–$SnBu_3$, $Ar^F$–H and unreacted $Ar^F$–I, was confirmed by $^{19}$F NMR.

**Synthesis of [Pd$I_2$(PN)]**

A mixture of *trans*-[Pd$Cl_2$(NCMe)$_2$] (55 mg, 0.212 mmol) and PN (67.7 mg, 0.212 mmol) in 10 mL of dry $CH_2Cl_2$ was stirred at room temperature for 1 h, and then n-hexane (20 mL) was added to afford a microcrystalline yellow solid which was washed with cold n-hexane (2 × 3 mL) and vacuum dried. The solid obtained, (*cis*-[Pd$Cl_2$(PN)]),[19] was dissolved in acetone/$CH_2Cl_2$ (10/10 mL) and KI (91.5 mg, 0.551 mmol) was added. The mixture was stirred for 1 h and then evaporated to dryness. The residue was extracted with 10 mL of dry $CH_2Cl_2$ and filtered through Celite. The solution obtained was evaporated to dryness giving a red solid which was washed with n-hexane (3 × 5 mL) and vacuum dried. Yield: 131 mg (91 %).

Anal. Calcd for $C_{21}H_{22}I_2NPPd$: C, 37.11; H, 3.26; N, 2.06. Found: C, 37.35; H, 3.42; N, 1.98.

<u>$^1$H NMR</u> (499.72 MHz, $CDCl_3$): δ 8.3–7.0 (m, 13H, $PPh_2$ + $C_6H_4$), 6.93 (m, 1H, $C_6H_4$), 4.1–2.3 (vbr, 8H, $CH_2$ + Me).

<u>$^1$H NMR</u> (499.72 MHz, $CDCl_3$, <u>233 K</u>): δ 8.2–7.2 (m, 13H, $PPh_2$ + $C_6H_4$), 6.92 (t, 1H, $^3J_{H\text{-}H}$ = 9.0 Hz, $C_6H_4$), 3.74 (d, 1H, $^2J_{H\text{-}H}$ = 13.0 Hz, $CH_2$), 3.29 (s, 3H, Me), 3.13 (m, 1H, $CH_2$), 2.74 (s, 3H, Me).

<u>$^{31}$P{$^1$H} NMR</u> (202.31 MHz, $CDCl_3$): δ 25.1 (s).

**Synthesis of [Pd(PN)($\eta^2$-dmfu)]**

The amino-phosphine PN (208 mg, 0.65 mmol) and dmfu (112 mg, 0.78 mmol) were added to a stirred suspension of $Pd_2(dba)_3$·$CHCl_3$ (673 mg, 0.65 mmol) in dry acetone (20 mL). Stirring was maintained for 4 h and the solution was evaporated to dryness giving a solid residue that was extracted with $CH_2Cl_2$ (20 mL) in the presence of activated charcoal. After filtration through Celite, the solution was first concentrated to a small volume (ca. 3 mL) and then diluted with diethyl ether to yield a yellow-orange solid which was filtered, washed with cold diethyl ether (2 × 5 mL) and vacuum dried. Yield: 296 mg (80 %).

Crystals valid for X-ray diffraction analysis were obtained by slow diffusion of n-hexane in a solution of the compound in $CH_2Cl_2$ (see molecular structure in Figure S7).

Anal. Calcd for $C_{27}H_{30}NO_4PPd$: C, 56.90; H, 5.31; N, 2.46. Found: C, 56.65; H, 5.13; N, 2.33.

$^1$H NMR (499.72 MHz, $CDCl_3$): δ 7.55–7.27 (m, 12H, $PPh_2$ + $C_6H_4$), 7.17 (m, 1H, $C_6H_4$), 6.83 (m, 1H, $C_6H_4$), 4.15 (dd, 1H, $^3J_{H\text{-}H}$ = 10.3 Hz, $J_{H\text{-}P}$ = 2.3 Hz, $CH_{dmfu}$), 3.79 (dd, 1H, $^3J_{H\text{-}H}$ = 10.3 Hz, $J_{H\text{-}P}$ = 10.3 Hz, $CH_{dmfu}$), 3.60 (s, 3H, $Me_{dmfu}$), 3.49 (d, 1H, $^2J_{H\text{-}H}$ = 12.4 Hz, $CH_2$), 3.23 (d, 1H, $^2J_{H\text{-}H}$ = 12.4 Hz, $CH_2$), 3.12 (s, 3H, $Me_{dmfu}$), 2.81 (s, 3H, $Me_{PN}$), 2.41 (s, 3H, $Me_{PN}$).

$^{31}P\{^1H\}$ NMR (202.31 MHz, $CDCl_3$): δ 21.3 (s).

## 3. Mechanistic support: Kinetic experiments

Weighed amounts of complex [Pd(C≡CPh)($SnBu_3$)(PN)] (**2**) (10.21 mg, 0.0125 mmol) and $Ar^F$–I (4.00 mg, 0.0123 mmol) were added inside a screw cap NMR tube with the aid of a Schlenk NMR tube adaptor along with a flame sealed coaxial capillary containing acetone-$d_6$ to lock the deuterium signal. The tube was cooled to 195 K in an isopropanol bath and subsequently, 0.50 mL of dry THF with dissolved trifluorotoluene as internal standard (0.025 mol × $L^{-1}$) were added with a microsyringe. The tube was closed inside the adaptor and then, taken out of the cool bath and mechanically shaken until total dissolution of the solids. Three experiments were carried out:

*a*) The reaction mixture was left either at 298 K for two days or at 323 K for 10 hours. The $^{19}$F NMR spectra recorded after that times are shown in Figures S1 (data collected in Scheme 2) and S2 respectively, confirming total conversion of $Ar^F$–I in both cases.

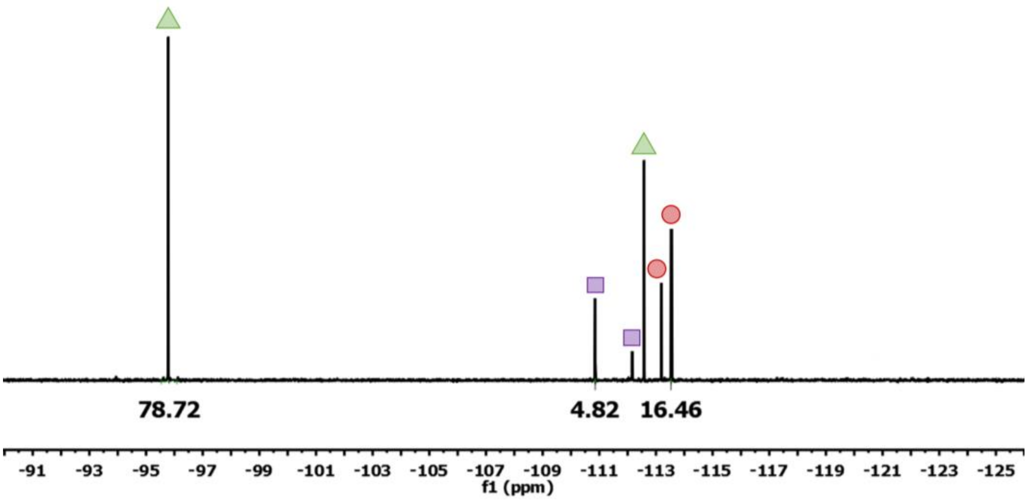


**Figure S1**. Final $^{19}$F NMR spectrum, with integrated $F^{ortho}$ signals, of the reaction of **2** with $Ar^F$–I in THF/ref-acetone-$d_6$ at 298 K. Green triangles correspond to $Ar^F$–$SnBu_3$, purple squares correspond to $Ar^F$–C≡CPh and red circles correspond to the $Ar^F$–H.

The $^{19}$F NMR shifts of the species potentially present in the mixture in THF/ref-acetone-$d_6$ are summarized below:

***$Ar^F$–I***; $^{19}$F NMR (470.17 MHz, THF/cap): δ –94.42 (2F, $F^o$), –115.23 (1F, $F^p$).

***$Ar^F$–$SnBu_3$***; $^{19}$F NMR (470.17 MHz, THF/cap): δ –95.79 (2F, $F^o$), –112.59 (1F, $F^p$).

***$Ar^F$–Alk***; $^{19}$F NMR (470.17 MHz, THF/cap): δ –110.86 (2F, $F^o$), –112.16 (1F, $F^p$).

***$Ar^F$–H***; $^{19}$F NMR (470.17 MHz, THF/cap): δ –113.20 (1F, $F^p$), –113.56 (2F, $F^o$).

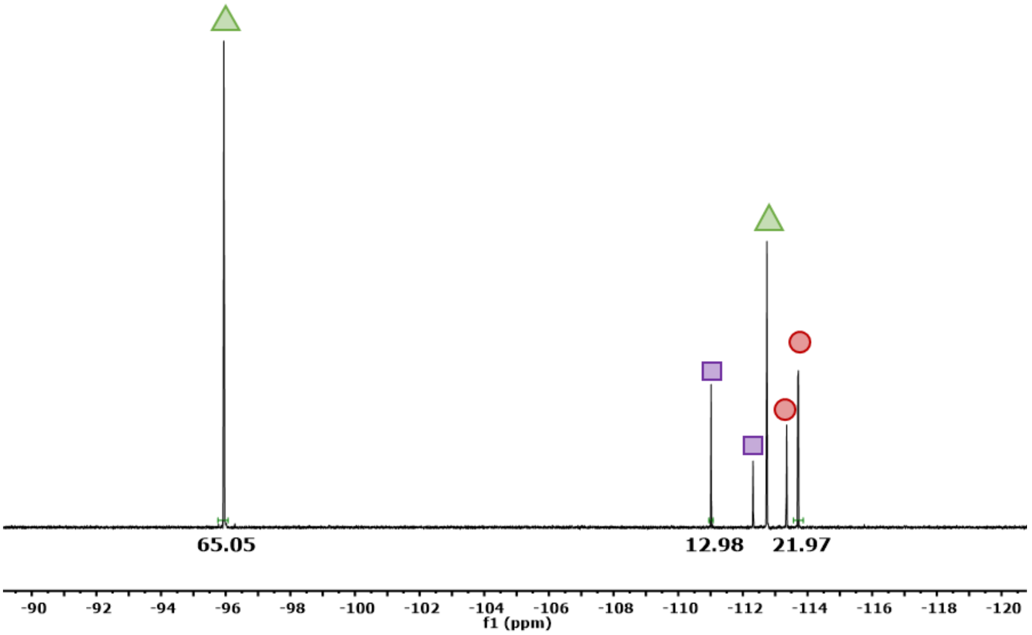


**Figure S2**. Final $^{19}F$ NMR spectrum, with integrated $F^{ortho}$ signals, of the reaction of **2** with $Ar^F$–I in THF/ref-acetone-$d_6$ at 323 K. Green triangles correspond to $Ar^F$–$SnBu_3$, purple squares correspond to $Ar^F$–C≡CPh and red circles correspond to the $Ar^F$–H.

*b*) The tube containing the reaction mixture was placed in a thermostated NMR probe at 283 K. Five minutes were left for temperature equilibration. Then, concentration-time data (triangles in Figure S3) were obtained from the integrals of the $F^{ortho}$ signals of the different species.

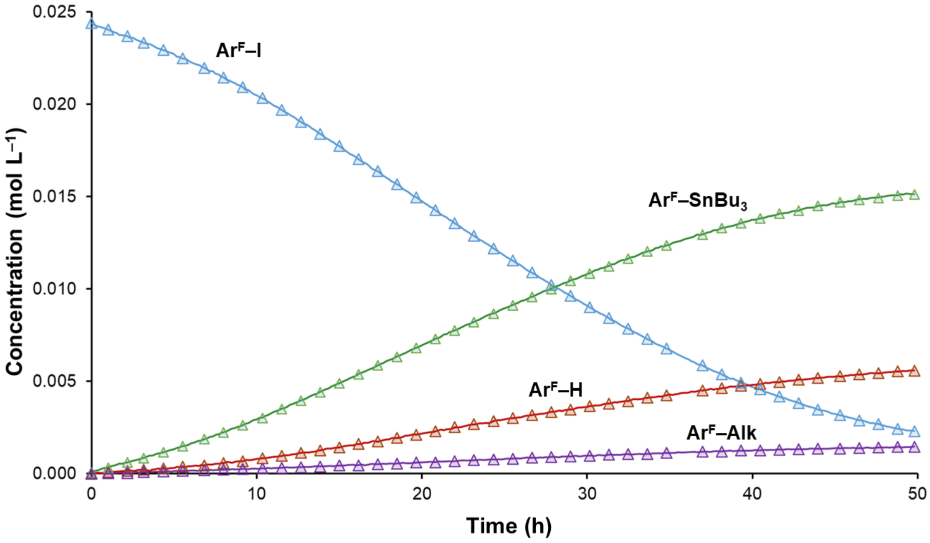


**Figure S3**. Concentration vs time data plot of experimental data (triangles) and COPASI-fitted values (continuous lines) of the fluorinated-species in the reaction of **2** with $Ar^F$–I in THF/ref-acetone-$d_6$ at 283 K.

The measured concentration *vs.* time experimental data were fitted (see continuous lines in Figure S3) by nonlinear least-squares (NLLS) regression, using the kinetic model depicted in Scheme S1 and the software COPASI.[20] Table S1 summarizes the adjusted kinetic constants. The refinement started using as initial guess the computed values for the first equilibrium ($k_1/k_{-1}$) given in the next section. The units of the rate constants are ($s^{-1}$) or ($mol^{-1} \times L \times s^{-1}$) for first and second order kinetic reactions respectively.

**Scheme S1**. Complete kinetic model, summarizing the plausible reaction pathways, to explain the competitive formation of $Ar^F$–$SnBu_3$, $Ar^F$–H and $Ar^F$–Alk.

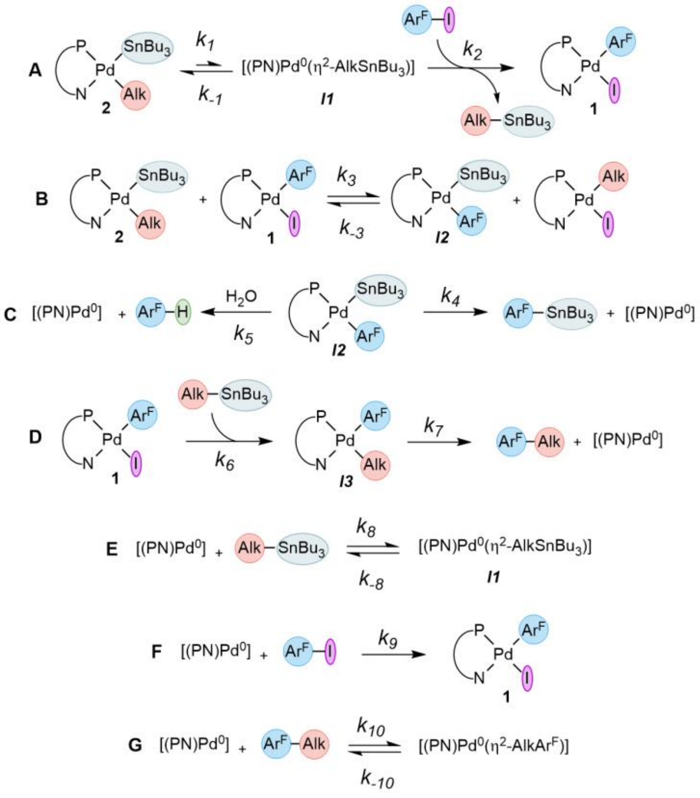


**Table S1.** Fitted rate constants. Starting conditions: [**2**]$_0$ = $2.50 \times 10^{-2}$ mol × L$^{-1}$; [Ar$^F$–I]$_0$ = 2.45 × $10^{-2}$ mol × L$^{-1}$; [$H_2O$]$_0$ = $6.00 \times 10^{-2}$ mol × L$^{-1}$. Best value of the fitting $6.82 \times 10^{-7}$.

| | |
|---|---|
| $\boldsymbol{k_1}$ | $2.800 \times 10^{-1}$ |
| $\boldsymbol{k_{-1}}$ | $1.173 \times 10^{3}$ |
| $\boldsymbol{k_2}$ | $5.408 \times 10^{-4}$ |
| $\boldsymbol{k_3}$ | $3.548 \times 10^{-1}$ |
| $\boldsymbol{k_{-3}}$ | $4.275 \times 10^{1}$ |
| $\boldsymbol{k_4}$ | $1.194 \times 10^{-1}$ |
| $\boldsymbol{k_5}$ | $7.172 \times 10^{-1}$ |

| | |
|---|---|
| $\boldsymbol{k_6}$ | $3.448 \times 10^{0}$ |
| $\boldsymbol{k_7}$ | $1.100 \times 10^{1}$ |
| $\boldsymbol{k_8}$ | $2.573 \times 10^{2}$ |
| $\boldsymbol{k_{-8}}$ | $1.244 \times 10^{0}$ |
| $\boldsymbol{k_9}$ | $2.113 \times 10^{-2}$ |
| $\boldsymbol{k_{10}}$ | $9.000 \times 10^{1}$ |
| $\boldsymbol{k_{-10}}$ | $5.499 \times 10^{0}$ |

## 4. Mechanistic support: Computational Studies

The density functional theory (DFT) calculations reported in this work were carried out using the dispersion corrected hybrid functional ωB97X-D developed by Head-Gordon and Chai,[21] and the Gaussian09 software.[22] The choice of this level of theory is based on the satisfactory results obtained in previous mechanistic studies with Sn derivatives.[23] C and H atoms were described using the double-ζ basis set 6-31G(d,p), whereas the same basis set plus diffuse functions was employed to describe the more electronegative N, Cl and F atoms. Pd, I and Sn atoms were described using the effective core potential LANL2DZ,[24] including f-polarization functions for Pd (exponent: 1.472),[25] or d-polarization functions for I and Sn (exponents: 0.289 and 0.180 respectively).[26]

Geometry optimizations in THF solution ($\varepsilon = 7.52$) using the SMD solvation model,[27] were performed without imposing any constraint, and the nature of the stationary points was further assessed through vibrational frequency analysis. As expected, all the energy minima were confirmed to display only real vibrational frequencies, whereas transition states were found to exhibit one single imaginary frequency. The latter were also confirmed to connect the expected energy minima by relaxing the transition state geometry along the reaction coordinate, following the eigenvector associated to the imaginary frequency. To simplify the calculations, $SnBu_3$ group was reduced to $SnMe_3$.

All the DFT data underlying this work, including the Cartesian coordinates of the modelled structures and energies, are available at the following ioChem-BD online data set: http://dx.doi.org/10.19061/iochem-bd-6-102

Scheme S2 summarizes additional results of our calculations. The free energy values for the $SnMe_3$ model fit, within 1 kcal mol$^{-1}$, the kinetic fitting obtained for the real molecules with $SnBu_3$, and confirm the thermodynamic preference of the oxidative additions of $Ar^F$–I and I-$SnMe_3$ with respect to the one of Alk–$SnMe_3$, leading to the formation of **1**, **3**$_{\mathbf{Me}}$ and **2**$_{\mathbf{Me}}$ respectively. The accessible activation barrier for reductive elimination from **2**$_{\mathbf{Me}}$ to intermediate ***I1***$_{\mathbf{Me}}$ (Figure 3, $\Delta G_{TS1} = 17.1$ kcal × mol$^{-1}$), confirms that the re-entrance of **2** into the classic Stille cycle is very easy. Moreover, the accessibility of the formation of the species [Pd($Ar^F$)($SnMe_3$)(PN)] (***I2***$_{\mathbf{Me}}$) from which the $Ar^F$–$SnMe_3$ coupling step is feasible also supports our mechanistic interpretation.

**Scheme S2**. Computed Gibbs energies, in THF solution, relevant for the proposed pathways ($\Delta G_0$ and $\Delta G^{\ddagger}$ values in kcal × mol$^{-1}$).

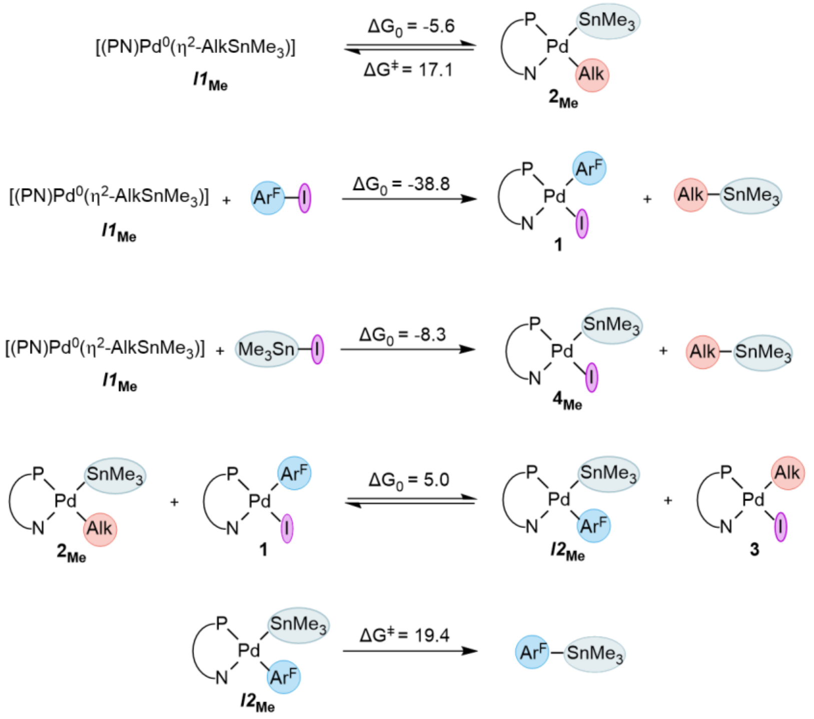


Figure S4 shows the computed structures of ***I1***$_{Me}$ and the ***TS1***$_{Me}$ to Alk–$SnMe_3$ reductive elimination. The substantial dissociation of the N atom of the PN ligand (2.910 Å) observed in ***TS1***$_{Me}$ explains the catalytic efficacy of hemilabile PN ligands compared to the poor performance of phosphines and diphosphines, mentioned in the text: PN ligands make the oxidative addition of $AlkSnBu_3$ to $Pd^0$(PN) an easily reversible process, which leads the evolution of the reaction to select the irreversible oxidative addition of $Ar^F$I. ***I1***$_{Me}$ features a triangular planar structure typical of alkynyl $Pd^0$ derivatives.[28]

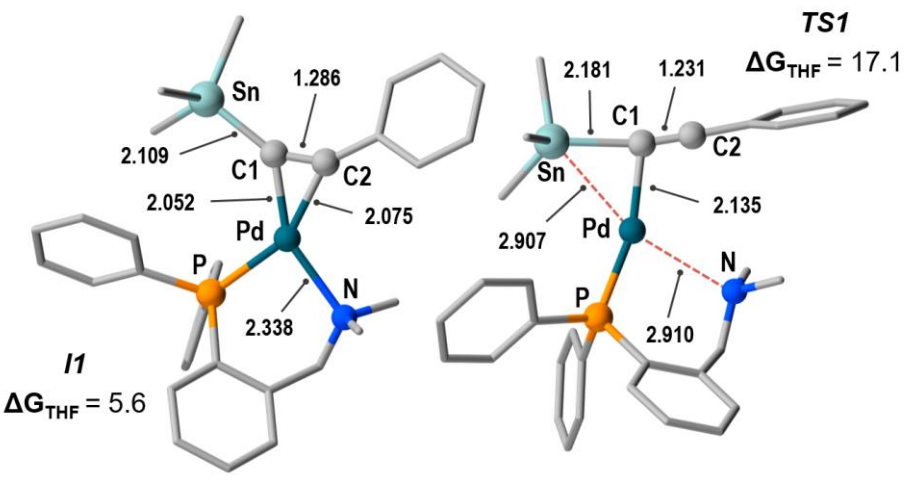


**Figure S4**. Optimized structures of ***I1***$_{Me}$ (left) and ***TS1***$_{Me}$ (right). Selected distances (in Å), $\Delta G_{THF}$ values in kcal × mol$^{-1}$.

Figure S5 gathers the optimized structures of ***I2$_{Me}$*** and the transition state for the subsequent Ar$^{F}$–SnMe$_3$ reductive elimination (***TS2$_{Me}$***).

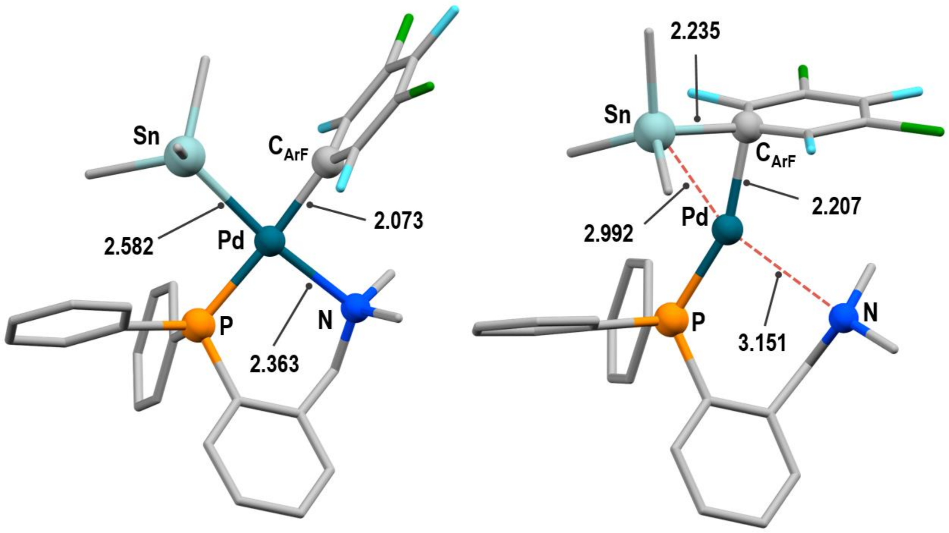


**Figure S5**. Optimized structures of ***I2$_{Me}$*** (left) and ***TS2$_{Me}$*** (right) with relevant distances in Å.

## 5. Catalytic experiments

### Reaction Conditions (Table 1 & S2)

In a glovebox, the corresponding palladium complex (0.02 or 0.004 mmol), tributyl(phenylethynyl)tin (75 µl, 0.20 mmol), $Ar^F$–I (65.0 mg, 0.20 mmol) and 2 mL of dry THF were added to an oven-dried Schlenk equipped with a spinvane Teflon stir bar. Substoichiometric $AsPh_3$ (10 mol% with respect to Pd, except in entry 11) and/or stoichiometric salts with respect to Sn (LiCl or CsF), were also added in certain experiments (see additives column in Table S2). For experiments 14-15, 0.20 mmol of 4-fluoroiodobenzene (instead of $Ar^F$–I) and 0.22 mmol of Alk–$SnBu_3$ were used.

The reaction was capped, removed from the glovebox and stirred at 323 K for 24 h. Then, the reaction mixture was cooled to room temperature and the conversion percentages gathered in Table S2 were calculated by $^{19}$F NMR.

**Table S2.** Catalytic results in the reaction of $Ar^F$–I ($Ar^F$ = $C_6F_3Cl_2$-3,5) with Alk–$SnBu_3$ (Alk = C≡CPh). Conversion percentages of each product.

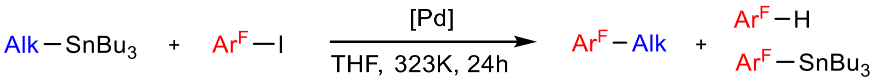


| Entry | Catalyst | Additives (mol%) | $Ar^F$Alk | $Ar^F$SnBu$_3$ | $Ar^F$H |
|---|---|---|---|---|---|
| 1 | **2** (10 %) | - | 50 | 28 | 22 |
| 2 | **1** (10 %) | - | 58 | 33 | 9 |
| 3 | **2** (10 %) | 1% $AsPh_3$ | 85 | 6 | 9 |
| 4 | **1** (10 %) | 1% $AsPh_3$ | 88 | 2 | 10 |
| 5 | **1** (10 %) | 10% $AsPh_3$ | 79 | - | 1 |
| 6 | **4** (10 %) | - | <1 | - | 5 |
| 7 | **1** (10 %) | 100% LiCl | 51 | 18 | 31 |
| 8 | **2** (10 %) | 1% $AsPh_3$, 100% LiCl | 90 | - | 10 |
| 9 | **1** (10 %) | 1% $AsPh_3$, 100% LiCl | **>99**[a] | - | - |
| 10 | **1** (2 %) | 0.2% $AsPh_3$ | 39 | 3 | 13 |
| 11 | **1** (2 %) | 0.2%$AsPh_3$, 100% LiCl | **96** | - | 4 |
| 12 | **1** (10 %) | 1% $AsPh_3$, 110% LiCl | **98**[b] | 2 | 0 |

[a] Analogous results are obtained for the reaction at 40 °C after 48 h (98%) or replacing LiCl with CsF. [b] Reaction with 4-$FC_6H_4I$. 110 mol% of Alk–$SnBu_3$ and LiCl are used because the first turnover from 1 can form up to 10% of PhC≡C-$C_6F_3Cl_2$-3,5 instead of PhC≡C–$C_6H_4F$-4.

**Characterization of the products**

The main compounds, $Ar^F$–C≡CPh, and $Ar^F$–$SnBu_3$, were synthesized following the reported procedures and fully characterized by NMR (see spectra in section 7), in order to unambiguously confirm their identity in the stoichiometric and catalytic experiments.

- ***$Ar^F$–C≡CPh***

$^1$H NMR (499.72 MHz, $CDCl_3$): δ 7.57 (m, 2H, Ph), 7.44–7.35 (m, 3H, Ph).

$^{19}$F NMR (470.16 MHz, $CDCl_3$): δ –108.44 (s, $2F^o$), –109.81 (s, $1F^p$).

$^{13}$C{$^1$H} NMR (125.67 MHz, $CDCl_3$): δ 157.2 (ddd, $^1J_{C–F}$ = 255.4 Hz, $^3J_{C–F}$ = 6.5 Hz, $^3J_{C–F}$ = 4.3 Hz, $CF^o$), 154.8 (dt, $^1J_{C–F}$ = 254 Hz, $^3J_{C–F}$ = 4.9 Hz, $CF^p$), 131.8 (s, $CH^o$), 129.5 (s, $CH^p$), 128.5 (s, $CH^m$), 121.7 (s, $C^{ipso}$Ph), 107.5 (m, *C*Cl), 101.0 (m, Ph–*C*≡C), 100.9 (td, $^2J_{C–F}$ = 21.0 Hz, $^4J_{C–F}$ = 4.5 Hz, $C^{ipso}Ar^F$), 73.5 (d, $^5J_{C–F}$ = 3.0 Hz, C≡*C*–$Ar^F$).

$^{13}$C{$^{19}$F} NMR was used to confirm the assignment of the signals corresponding to the $Ar^F$ group.

- ***$Ar^F$–$SnBu_3$***

$^1$H NMR (499.72 MHz, $CDCl_3$): δ 1.52 (m, 6H, $SnCH_2CH_2$), 1.33 (m, 6H, $CH_2CH_3$), 1.22 (m, 6H, $SnCH_2$), 0.90 (t, 9H, *J* = 7.3 Hz, $CH_3$).

$^{19}$F NMR (470.16 MHz, $CDCl_3$): δ –94.59 (br, $2F^o$), –110.54 (t, $J_{F–F}$ = 2.8, $1F^p$).

$^{119}$Sn{$^1$H} NMR (186.35 MHz, $CDCl_3$): δ –18.3 (dt, $^5J_{F–119Sn}$ = 6.4 Hz; $^3J_{F–119Sn}$ = 3.4 Hz).

$^{13}$C{$^1$H} NMR (125.67 MHz, $CDCl_3$): δ 160.1 (ddd, $^1J_{C–F}$ = 238.2 Hz, $^3J_{C–F}$ = 22.4 Hz, $^3J_{C–F}$ = 5.0 Hz, $CF^o$), 155.5 (dt, $^1J_{C–F}$ = 250.2 Hz, $^3J_{C–F}$ = 5.5 Hz, $CF^p$), 110.6 (td, $^2J_{C–F}$ = 53.8 Hz, $^4J_{C–F}$ = 3.6 Hz, $C^{ipso}$), 106.2 (ddd, $^2J_{C–F}$ = 30.0 Hz, $^2J_{C–F}$ = 20.6 Hz, $^4J_{C–F}$ = 5.9 Hz, *C*Cl), 28.8 (s, sat. $^3J_{C–Sn}$ = 21.0 Hz, $CH_2CH_3$), 27.1 (s, sat. $^2J_{C–119Sn}$ = 65.8 Hz, sat. $^2J_{C–117Sn}$ = 63.0 Hz, $SnCH_2CH_2$), 13.5 (s, sat. $^4J_{C–Sn}$ = 2.4 Hz, $CH_3$), 11.5 (t, $^4J_{C–F}$ = 2.0 Hz, sat. $^1J_{C–119Sn}$ = 358.6 Hz, sat. $^1J_{C–117Sn}$ = 343.0, $SnCH_2$).

$^{13}$C{$^{19}$F} NMR was used to confirm the assignment of the signals corresponding to the $Ar^F$ group.

* Note that $Ar^F$–H and PhC≡C–$C_6H_4$F-4 have been previously reported (see General Information section). ** $^{31}$P NMR spectrum after complete conversion in the experiment in entry 1 confirms the presence of complexes **2**, **3** and **4**. Mass spectrometry also reveals the formation of the homocoupling product PhC≡C–C≡CPh.[29]

## Reaction Conditions (Table 2 & S3)

In a glovebox, the corresponding palladium complex (0.02 or 0.004 mmol), tributyl(phenylethynyl)tin (75 µl, 0.20 mmol), $Ar^F$–I (0.20 mmol) and 2 mL of dry THF were added to an oven-dried Schlenk equipped with a spinvane Teflon stir bar. Substoichiometric $AsPh_3$ (10 mol% with respect to Pd) and stoichiometric LiCl with respect to Sn, were also added. The reaction was capped, removed from the glovebox and stirred at 323 K for 24 h. Then, the reaction mixture was cooled to room temperature and the conversion percentages gathered in Table S3 were calculated by $^{19}F$ NMR.

This procedure is selected following the reaction conditions that offered the best results in Table 1.

**Table S3.** Catalytic $Ar^F$–Alk results of $Ar^F$–I + PhC≡C–$SnBu_3$ coupling catalyzed by complex **1**, with different fluorinated aryl groups.

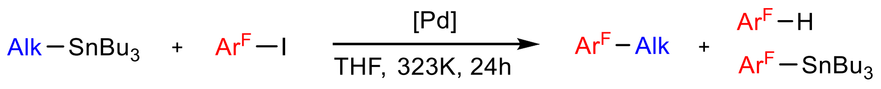


| Entry | $Ar^FI$ | Catalyst | $Ar^FI$ | $Ar^FAlk$ | $Ar^FH$ |
|---|---|---|---|---|---|
| 1[a] | 3,5-$C_6F_3Cl_2I$ | **1** (10%) | 0 | **>99** | 0 |
| 2[b] | 4-$FC_6H_4I$ | **1** (10%) | 0 | **98**[c] | 0 |
| 3 | 2-$FC_6H_4I$ | **1** (10%) | 11 | 82 | 7 |
| 4 | 2,6-$F_2C_6H_3I$ | **1** (10%) | 70 | 30 | 0 |
| 5[d] | 2,6-$F_2C_6H_3I$ | **1** (10%) | 10 | 70 | 10[e] |
| 6 | $C_6F_5I$ | **1** (10%) | 4 | 70 | 26 |
| 7[f] | $C_6F_5I$ | **1** (2%) | 3 | 70 | 27 |

[a] Entry 9 of Table 1. [b] Entry 12 of Table 1. [c] 2% $Ar^FSnBu_3$. [d] 12 h at 100 °C. [e] Plus others (10%). [f] 24 h 90 °C 1,4-Dioxane.

**Reaction Conditions (Table 3 & S4)**

In a glovebox, the corresponding palladium complex (0.02 or 0.004 mmol), Ph–C≡C–X ( X = Cl, I) (0.20 mmol), $Ar^F$–$SnBu_3$ (98.0 mg, 0.20 mmol) and 2 mL of dry THF were added to an oven-dried Schlenk equipped with a spinvane Teflon stir bar. Substoichiometric $AsPh_3$ (10 mol% with respect to Pd, when indicated) and/or stoichiometric LiCl with respect to Sn were also added in certain experiments (see additives column in Table S3).

The reaction was capped, removed from the glovebox and stirred at 323 K for 24 h. Then, the reaction mixture was cooled to room temperature and the conversion percentages gathered in Table S3 were calculated by $^{19}$F NMR. For experiment in entry 5, 0.22 mmol of Ph–C≡C–Cl were used instead of Ph–C≡C–I and the reaction was stirred for 36 h at 343 K.

**Table S4.** Catalytic results in the reaction of Ph–C≡C–X (X = I; Cl) with $Ar^F$–$SnBu_3$. Conversion percentages of each product.

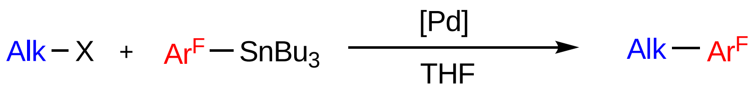


| Entry | Catalyst | Additives [a] | $Ar^F$–Alk | $Ar^F$–$SnBu_3$ | $Ar^F$–H |
|---|---|---|---|---|---|
| 1 | **3** (10 %) * | - | 81 | 18 | 1 |
| 2 | **3** (10 %) *,a,b | $AsPh_3$, LiCl | >99 | - | - |
| 3 | **3** (10 %) *,b | LiCl | >99 | - | - |
| 4 | **3** (2 %) *,a | $AsPh_3$ | 68 | 29 | 3 |
| 5 | **3** (2%) *,b | LiCl | **98** | - | 2 |
| 6 | **5** (2%) ** | - | 31 | 65 | 4 |
| 7 | **5** (2%) **,a | $AsPh_3$ | **97** | - | 3 |
| 8 | **2** (10 %) ** | - | 21 | 51 | 28 |
| 9 | **2** (10 %) **,a | $AsPh_3$ | 89 | 6 | 5 |
| 11 | **2** (2 %) **,a | $AsPh_3$ | 82 | 12 | 6 |

[a] Substoichiometric $AsPh_3$ (10 mol% with respect Pd catalyst). [b] Stoichiometric LiCl (100 mol %). *Alk–I is used as reactant and the reaction is left for 24 h at 50 ºC. **Alk–Cl is used as reactant and the reaction is left for 36 h at 70 ºC.

**Reaction Conditions (Table 4 & S5)**

In a glovebox, complex **5** (0.02 or 0.004 mmol), Ph–C≡C–Cl (0.20 mmol), $C_6F_5$–$SnBu_3$ (0.20 mmol), substoichiometric $AsPh_3$ (10 mol% with respect to Pd, when indicated) and 2 mL of dry 1,4-Dioxane were added to an oven-dried Schlenk equipped with a spinvane Teflon stir bar.

The reaction was capped, removed from the glovebox and stirred at the indicate temperature for 24 or 36 h. Then, the reaction mixture was cooled to room temperature and the conversion percentages gathered in Table S5 were calculated by $^{19}F$ NMR using trifluorotoluene as internal standard.

**Table S5.** Catalytic results of $C_6F_5$–Alk couplings using Alk–Cl, and $C_6F_5$–$SnBu_3$, catalyzed by 5. SM = starting materials.

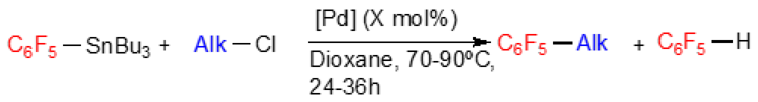


| Entry | t (h) | T ºC | Catalyst | SM | $C_6F_5$-Alk | $C_6F_5$-H |
|---|---|---|---|---|---|---|
| 1[a] | 24 | 70 | 5 (10%) | 32 | 61 | 7 |
| 2[a] | 36 | 70 | 5 (10%) | 0 | **87** | 13 |
| 3[a] | 36 | 90 | 5 (2%) | 21 | 72 | 7 |

[a] Substoichiometric $AsPh_3$ (10 mol% with respect Pd catalyst).

**Reaction Conditions (Table 5 & S6)**

In a glovebox, complex [$PdCl_2$(Ph-PEWO-F)] (0.01 mmol) or (*trans*-[$PdCl_2(NCMe)_2$] (0.02 mmol) and tBuXPhos (0.02 mmol), R–X (0.20 mmol), R'–$SnBu_3$ and 2 mL of dry 1,4-Dioxane were added to an oven-dried Schlenk equipped with a spinvane Teflon stir bar.

The reaction was capped, removed from the glovebox and stirred at 353 K for 24 h. Then, the reaction mixture was cooled to room temperature and the conversion percentages gathered in Table S6 were calculated by $^{19}F$ NMR using trifluorotoluene as internal standard.

**Table S6.** Catalytic results of $C_6F_5$–Alk couplings using $C_6F_5$–I + $Bu_3Sn$–C≡CPh, or the transposed combination using Alk–Cl, catalyzed by [$PdCl_2$(Ph-PEWO-F)] (**6**) or {[$PdCl_2(CH_3CN)_2$] + tBuXPhos} (**7**). SM = starting materials.

| Entry | R′–Sn | R–X | Cat | SM | R–R′ | $C_6F_5H$ |
|---|---|---|---|---|---|---|
| 1[a,b] | PhCC–Sn | $C_6F_5$–I | **6** (5%) | 5 | **85** | 6* |
| 2[b] | $C_6F_5$–Sn | PhCC–Cl | **6** (5%) | 30 | 30 | 40 |
| 3[a,c] | PhCC–Sn | $C_6F_5$–I | **7** (10%) | <1 | **86** | 14 |
| 4[c] | $C_6F_5$–Sn | PhCC–Cl | **7** (10%) | 58 | 1 | 41 |

* 4% of unknown Pd($C_6F_5$) species. R–X (1 equiv) [a] Stoichiometric LiCl (100 mol %). [b] R′–$SnBu_3$ (1.1 equiv). [c] R′–$SnBu_3$ (1.2 equiv).

## Reaction Conditions (Table 6 & S7)

In a glovebox, complex **2** (0.02 or 0.004 mmol), $^t$Bu–C≡C–I (41.6 mg, 0.20 mmol), tributyl(phenylethynyl)tin (75 µl, 0.20 mmol) and 2 mL of dry THF were added to an oven-dried Schlenk equipped with a spinvane Teflon stir bar. Substoichiometric $AsPh_3$ (10 mol% with respect to Pd, when indicated) and/or stoichiometric LiCl with respect to Sn were also added in certain experiments (see additives column in Table S4).

The reaction was capped, removed from the glovebox and stirred at 323 K for 24 h. Then, the reaction mixture was cooled to room temperature and the conversion percentages gathered in Table S4 were calculated by mass spectrometry.

**Table S7.** Catalytic results in the reaction of $^t$Bu–C≡C–I with PhC≡C–$SnBu_3$. Conversion percentages of each product.

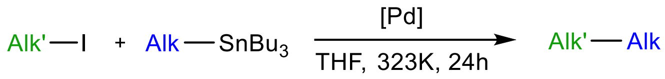


| Entry | Catalyst | Additives[a] | Alk′–Alk | Alk′–Alk′ | Alk′–I |
|---|---|---|---|---|---|
| 1 | **2** (10 mol%) | $AsPh_3$ LiCl | **92** | 8 | <1 |
| 2 | **2** (10 mol%) | LiCl | **92** | 8 | <1 |
| 3 | **2** (10 mol%) | - | **91** | 9 | <1 |
| 4 | **2** (2 mol%) | LiCl | **91** | 9 | <1 |
| 5 | **2** (2 mol%) | - | 58 | 6 | 36 |

[a] Substoichiometric $AsPh_3$ (10 mol% with respect Pd catalyst). * Note that the unsymmetrical 1,3-dyine PhC≡C–C≡C$^t$Bu has been previously reported (see General Information section). $^t$BuC≡C–C≡C$^t$Bu (Alk′–Alk′ in the table) has also been reported.[30]

## 6. X-ray diffraction details

Refinement of the X-ray structures gives the residuals shown in Table S5.

A crystal was attached to a glass fiber and transferred to an Agilent Supernova diffractometer with an Atlas CCD area detector. Data collection was performed with Mo-Kα radiation (λ = 0.71073 Å). Data integration, scaling and empirical absorption correction was carried out using the CrysAlisPro program package.[31] The crystal was kept at 294 or 180 K during data collection. Using Olex2,[32] the structure was solved with the ShelxT,[33] and refined with Shelx program.[34] The non-hydrogen atoms were refined anisotropically and hydrogen atoms were placed at idealized positions and refined using the riding model. CCDC 2108467, 2108468, 2154510 and 2211180. contain the supporting crystallographic data for this paper. These data can be obtained free of charge at www.ccdc.cam.ac.uk/conts/retrieving.html [or from the Cambridge Crystallographic Data Centre, 12, Union Road, Cambridge CB2 1EZ, UK; fax: (internat.) +44-1223/336-033; E-mail: deposit@ccdc.cam.ac.uk]. Figures 1, 4 and S6 show the molecular structures of complexes **2**, **3** and **5** respectively.

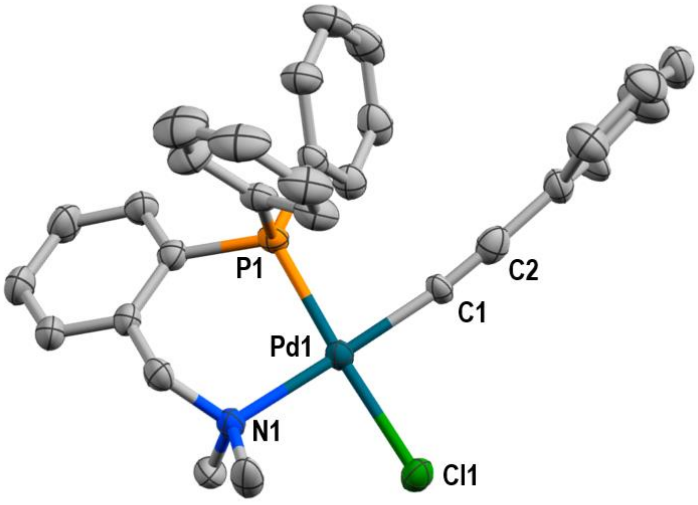


**Figure S6**. X-Ray structure of **5**. Hydrogen atoms have been omitted for clarity. Relevant distances (Å) and angles (°): Pd1–Cl1 = 2.3701(9), Pd1–P1 = 2.2220(9), Pd1–N1 = 2.160(2), Pd1–C1 = 1.990(3); C1–C2 = 1.142(4); C1–Pd1–Cl1 = 90.20(9), P1–Pd1–N1 = 92.77(7), P1–Pd1–C1 = 86.02(9), Cl1–Pd1–N1 = 91.61(7).

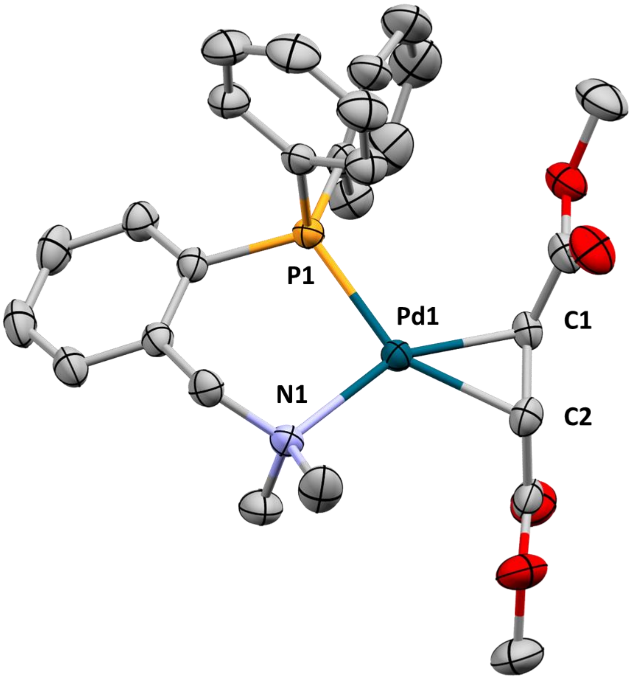


**Figure S7**. X-Ray structure of **[Pd(PN)(η$^2$-dmfu)]**. Hydrogen atoms have been omitted for clarity. Relevant distances (Å) and angles (°): Pd1–P1 = 2.2616(12), Pd1–N1 = 2.199(4), Pd1–C1 = 1.990(3); Pd1–C1 = 2.061(4), Pd1–C2 = 2.130(5); C1–Pd1–C2 = 39.50(18), P1–Pd1–N1 = 94.13(10), P1–Pd1–C1 = 111.63(14), C2–Pd1–N1 = 154.18(17).

**Table S7.** Crystal data and structure refinements for complexes [Pd(C≡CPh)($SnBu_3$)(PN)] (**2**), [PdI($SnBu_3$)(PN)] (**4**) , [Pd(C≡CPh)Cl(PN)] (**5**) and [$Pd^0$(PN)($\eta^2$-dmfu)].

| | **2** | **4** | **5** | **[$Pd^0$(PN)($\eta^2$-dmfu)]** |
|---|---|---|---|---|
| Empirical formula | $C_{41}H_{54}NPPdSn$ | $C_{33}H_{49}INPPdSn$ | $C_{29}H_{27}ClNPPd$ | $C_{27}H_{30}NO_4PPd$ |
| Formula weight | 816.91 | 842.69 | 562.33 | 569.89 |
| Temperature/K | 180.05(10) | 294. | 294.0 | 295 |
| Crystal system | triclinic | monoclinic | monoclinic | monoclinic |
| Space group | P-1 | $P2_1/c$ | $P2_1/n$ | P21/c |
| a/Å | 11.5953(4) | 20.3642(10) | 12.5081(8) | 11.1284(7) |
| b/Å | 13.1129(5) | 10.6581(3) | 17.8986(7) | 13.8540(12) |
| c/Å | 14.2737(5) | 18.7632(7) | 12.8073(7) | 17.0553(11) |
| α/° | 89.074(3) | 90 | 90 | 90 |
| β/° | 88.178(3) | 116.691(5) | 114.934(7) | 96.943(6) |
| γ/° | 64.020(4) | 90 | 90 | 90 |
| Volume/Å$^3$ | 1949.98(14) | 3638.5(3) | 2600.0(3) | 2610.2(3) |
| Z | 2 | 4 | 4 | 4 |
| $\rho_{calc}$g/cm$^3$ | 1.391 | 1.538 | 1.437 | 1.45 |
| μ/mm$^{-1}$ | 1.170 | 2.093 | 0.895 | 0.804 |
| F(000) | 836.0 | 1672.0 | 1144.0 | 1168 |
| Crystal size/mm$^3$ | 0.745 × 0.447 × 0.133 | 0.416 × 0.259 × 0.238 | 0.574 × 0.246 × 0.162 | 0.415 × 0.235 × 0.169 |
| Radiation | MoKα (λ = 0.71073) | MoKα (λ = 0.71073) | MoKα (λ = 0.71073) | MoKα (λ = 0.71073) |
| 2θ range for data collection/° | 6.832 to 59.216 | 6.718 to 59.512 | 6.788 to 59.536 | 6.944 to 58.898 |
| Index ranges | $-14 \le h \le 15$, $-12 \le k \le 16$, $-19 \le l \le 19$ | $-27 \le h \le 27$, $-14 \le k \le 14$, $-18 \le l \le 26$ | $-13 \le h \le 15$, $-24 \le k \le 16$, $-17 \le l \le 12$ | $-12 \le h \le 15$, $-12 \le k \le 18$, $-22 \le l \le 16$ |
| Reflections collected | 13487 | 25084 | 11285 | 11187 |
| Independent reflections | 8951 [$R_{int}$ = 0.0162, $R_{sigma}$ = 0.0379] | 8901 [$R_{int}$ = 0.0325, $R_{sigma}$ = 0.0446] | 6078 [$R_{int}$ = 0.0277, $R_{sigma}$ = 0.0513] | 6081 [Rint = 0.0353, Rsigma = 0.0657] |
| Data/restraints/parameters | 8951/3/448 | 8901/3/361 | 6125/0/318 | 6081/0/311 |
| Goodness-of-fit on F$^2$ | 1.068 | 1.021 | 1.065 | 1.077 |
| Final R indexes [I>=2σ (I)] | $R_1$ = 0.0315, $wR_2$ = 0.0661 | $R_1$ = 0.0458, $wR_2$ = 0.0904 | $R_1$ = 0.0389, $wR_2$ = 0.0769 | R1 = 0.0526, wR2 = 0.1083 |
| Final R indexes [all data] | $R_1$ = 0.0444, $wR_2$ = 0.0749 | $R_1$ = 0.0972, $wR_2$ = 0.1131 | $R_1$ = 0.0662, $wR_2$ = 0.0938 | R1 = 0.0932, wR2 = 0.1333 |
| Largest diff. peak/hole / eÅ$^{-3}$ | 0.45/-0.58 | 0.75/-0.68 | 0.53/-0.39 | 1.56/-0.69 |

# 7. NMR spectra: $^{1}H$, $^{13}C$, $^{19}F$, $^{31}P$ and $^{119}Sn$

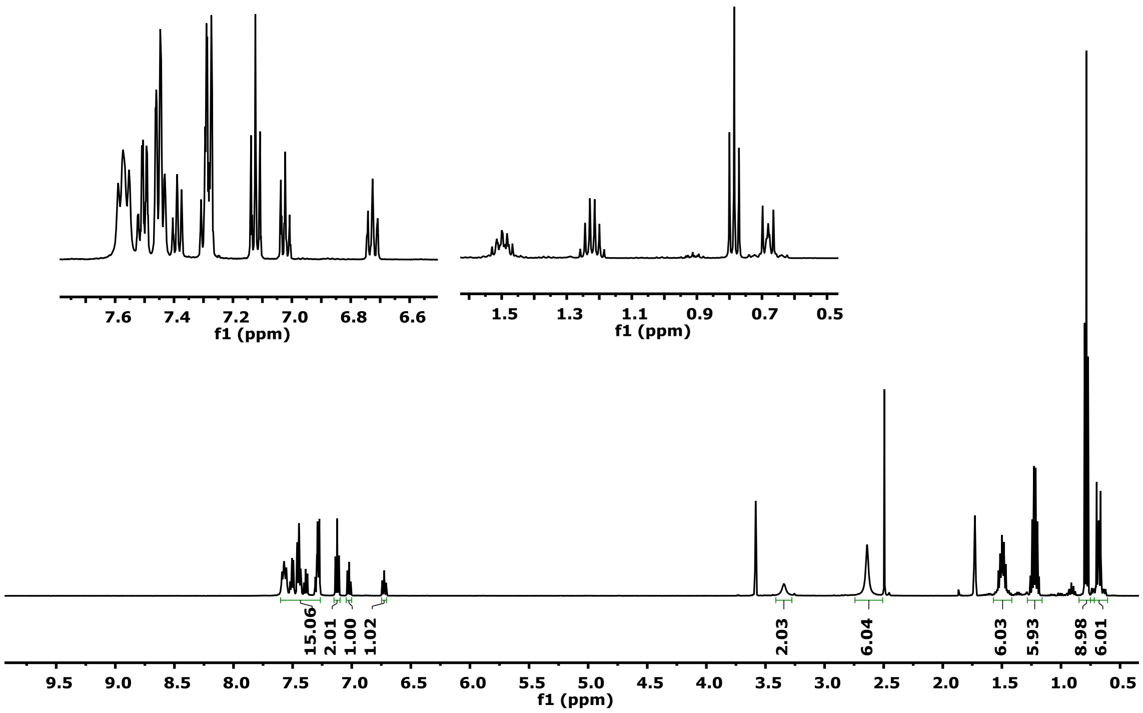


**Figure S8.** $^{1}H$ NMR (499.72 MHz, 298 K, THF-$d_8$) of [Pd(C≡CPh)($SnBu_3$)(PN)] (**2**).

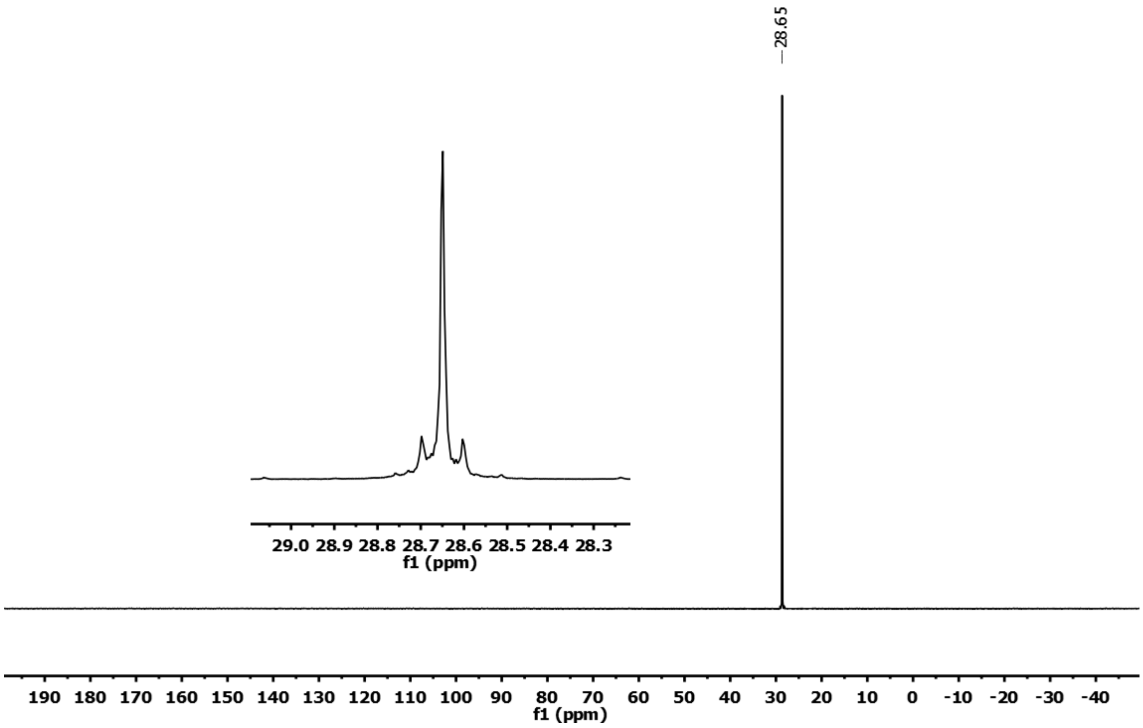


**Figure S9**. $^{31}P\{^{1}H\}$ NMR (202.31 MHz, 298 K, THF-$d_8$) of [Pd(C≡CPh)($SnBu_3$)(PN)] (**2**).

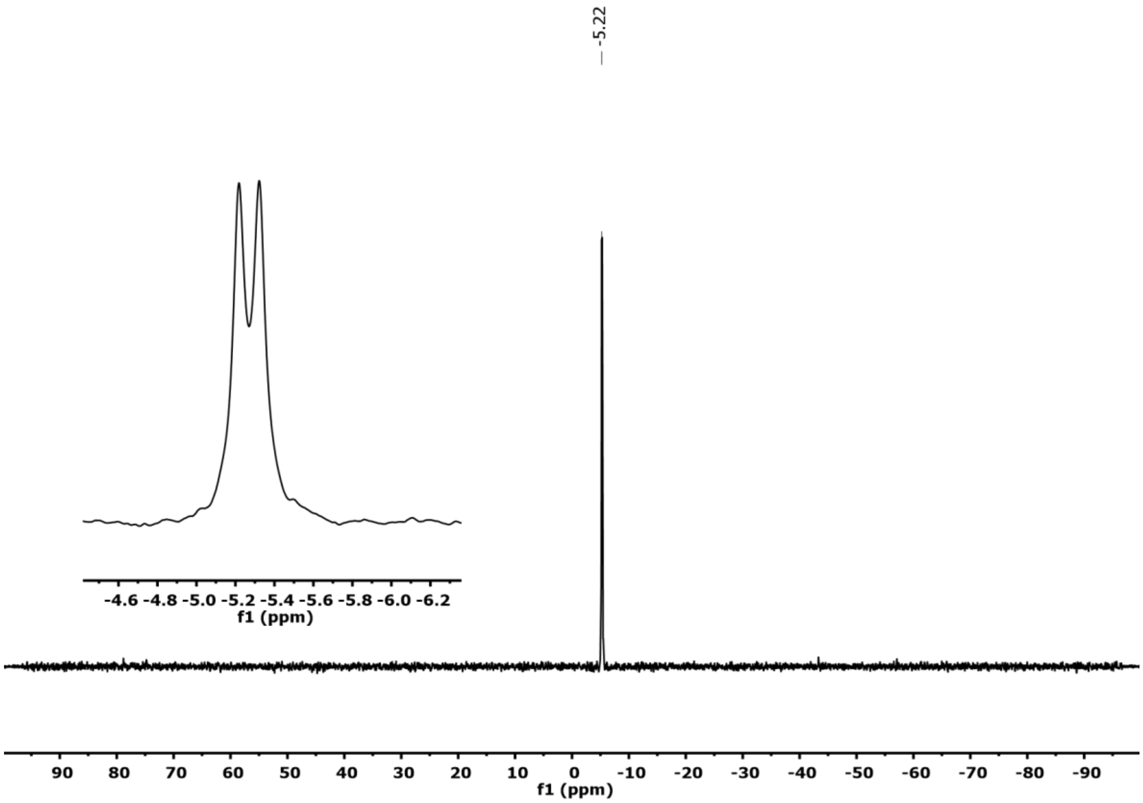


**Figure S10.** $^{119}Sn\{^{1}H\}$ NMR (186.35 MHz, 298 K, THF–$d_8$) of [Pd(C≡CPh)(SnBu$_3$)(PN)] (**2**).

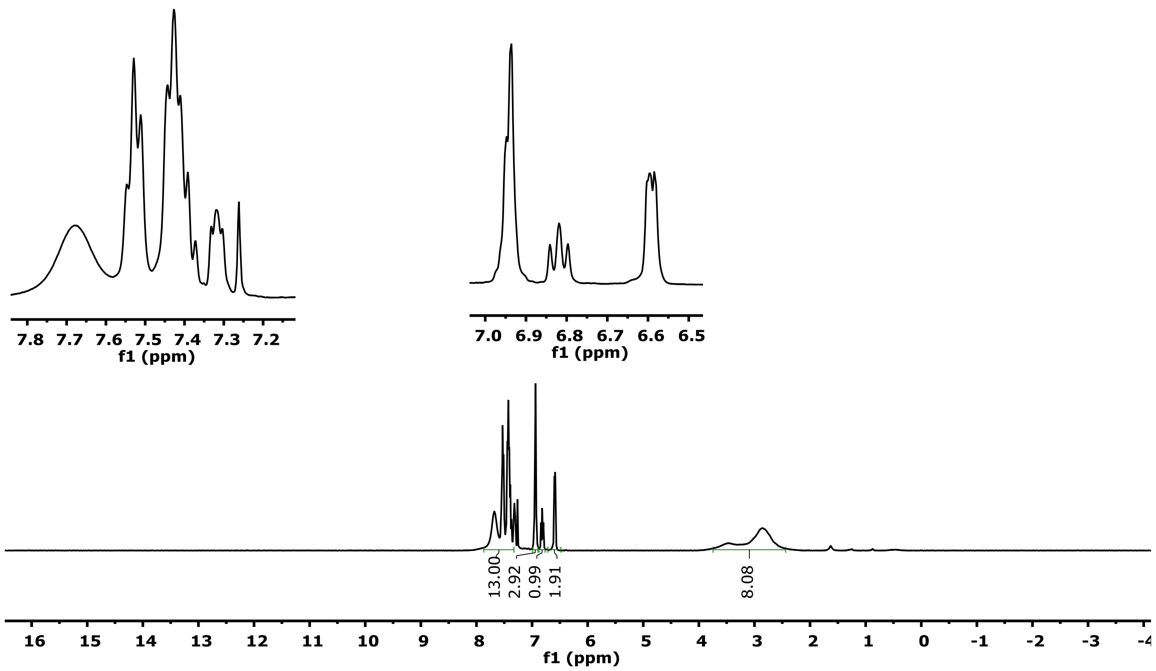


**Figure S11.** $^{1}H$ NMR (499.72 MHz, 298 K, $CDCl_3$) of [Pd(C≡CPh)I(PN)] (**3**).

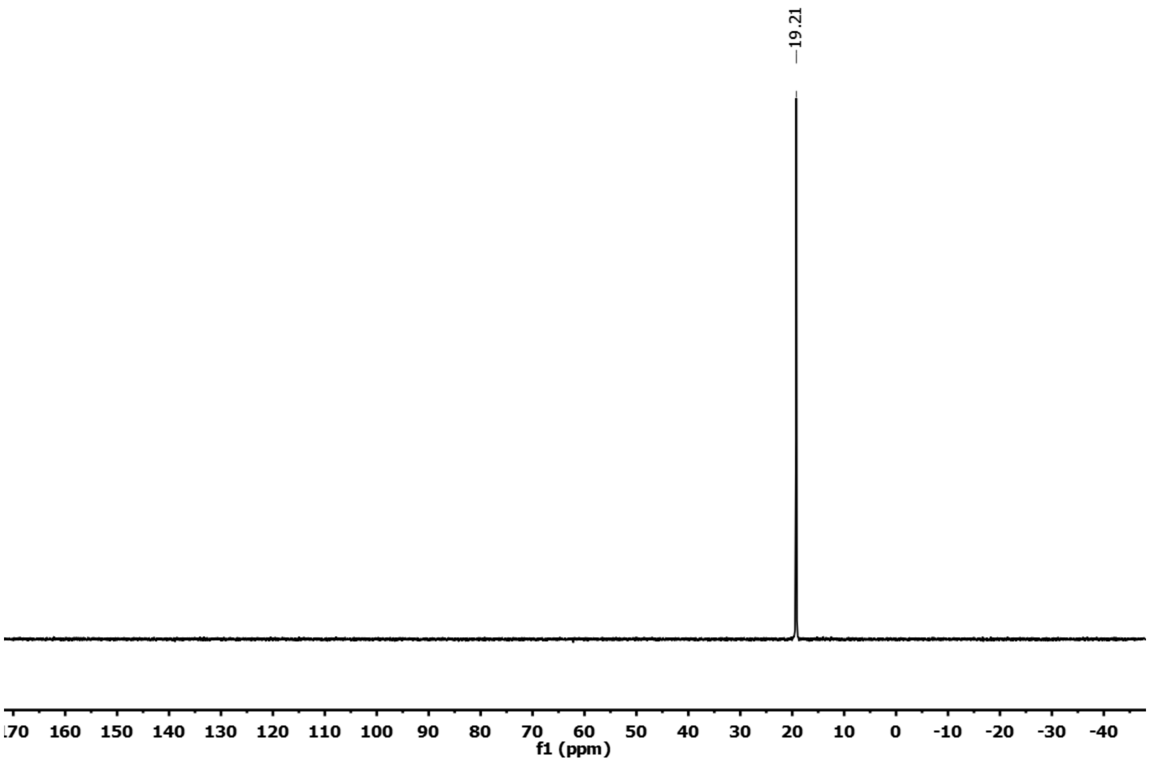


**Figure S12**. $^{31}P\{^{1}H\}$ NMR (202.31 MHz, 298 K, $CDCl_3$) of [Pd(C≡CPh)I(PN)] (**3**).

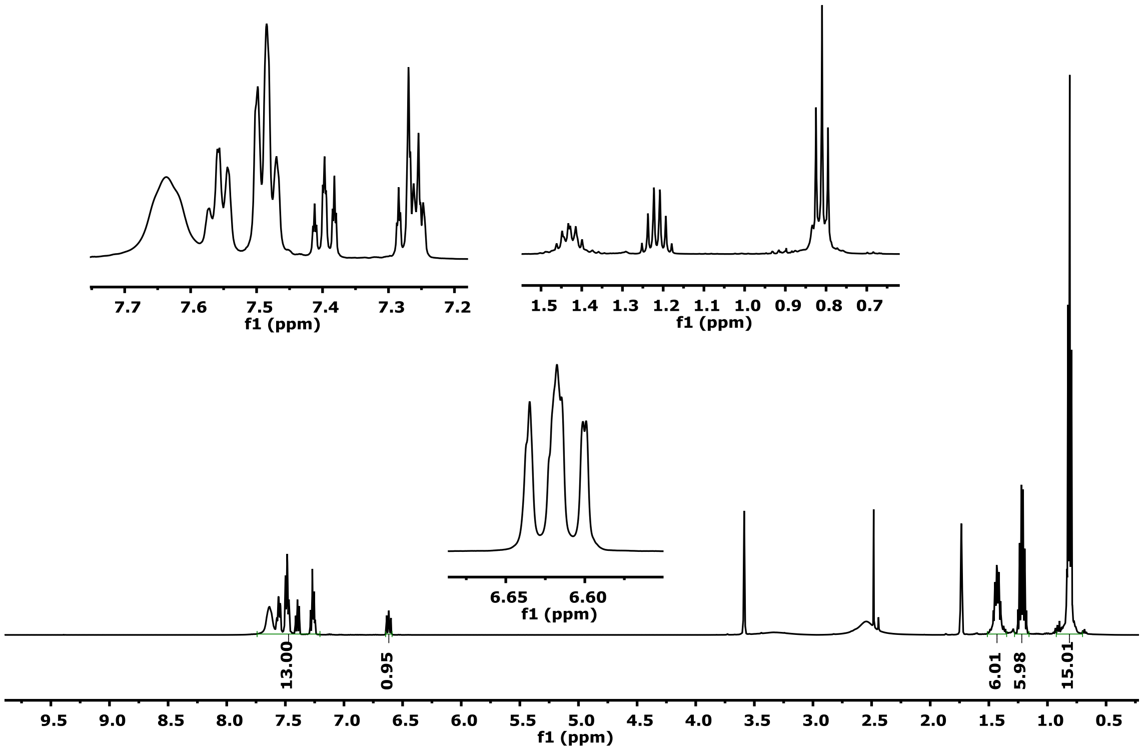


**Figure S13.** $^{1}H$ NMR (499.72 MHz, 298 K, THF-$d_8$) of [PdI($SnBu_3$)(PN)] (**4**).

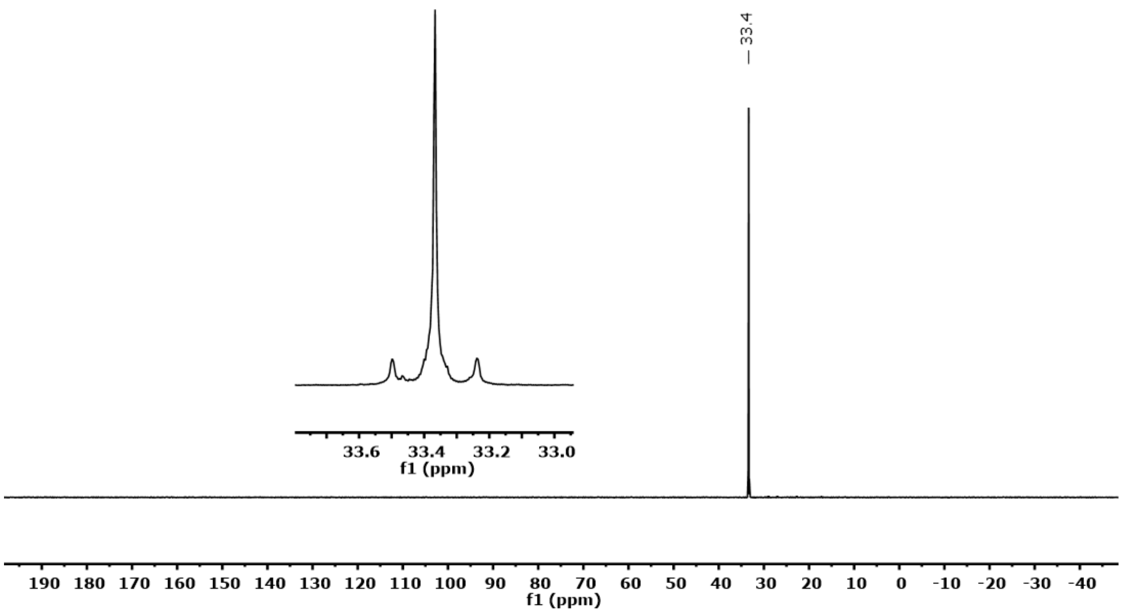


**Figure S14.** $^{31}P\{^{1}H\}$ NMR (202.31 MHz, 298 K, THF-$d_8$) of [PdI(SnBu$_3$)(PN)] (**4**).

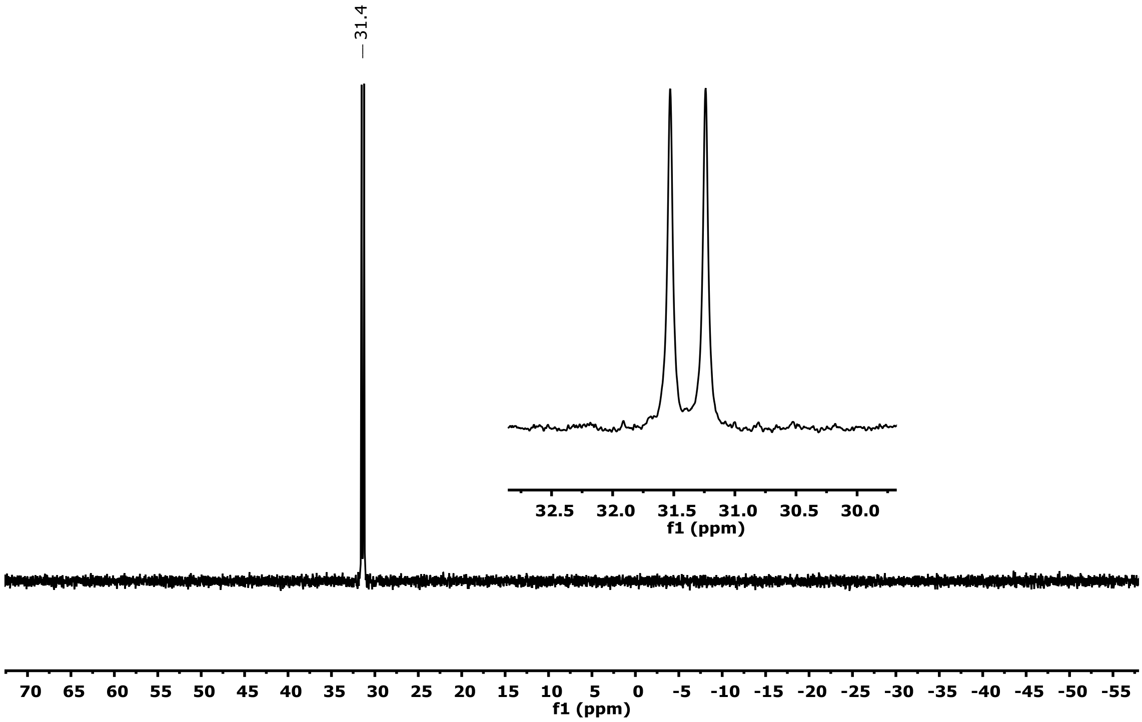


**Figure S15.** $^{119}Sn\{^{1}H\}$ NMR (186.35 MHz, 298 K, THF-$d_8$) of [PdI(SnBu$_3$)(PN)] (**4**).

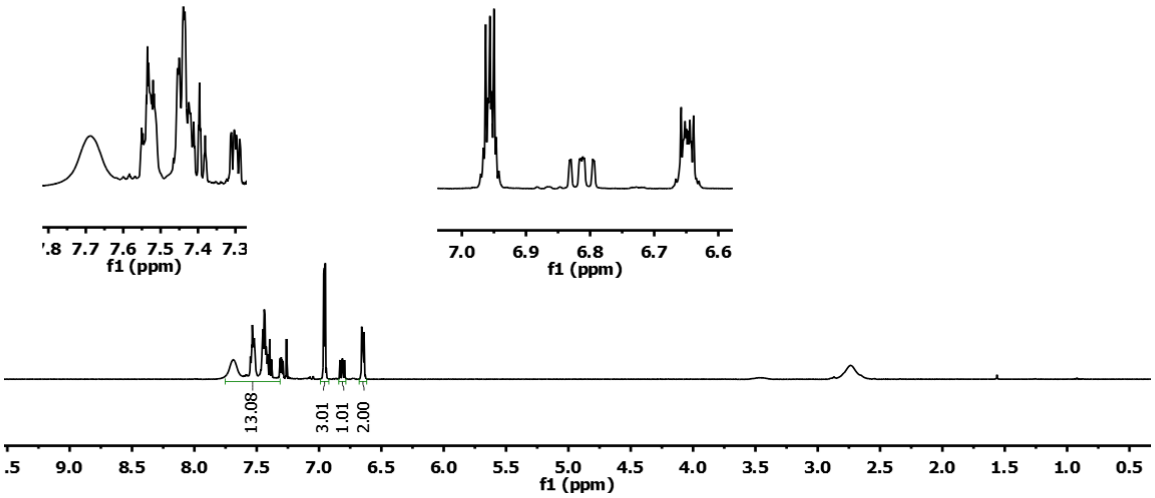


**Figure S16.** $^{1}H$ NMR (499.72 MHz, 298 K, $CDCl_3$) of [Pd(C≡CPh)Cl(PN)] (**5**).

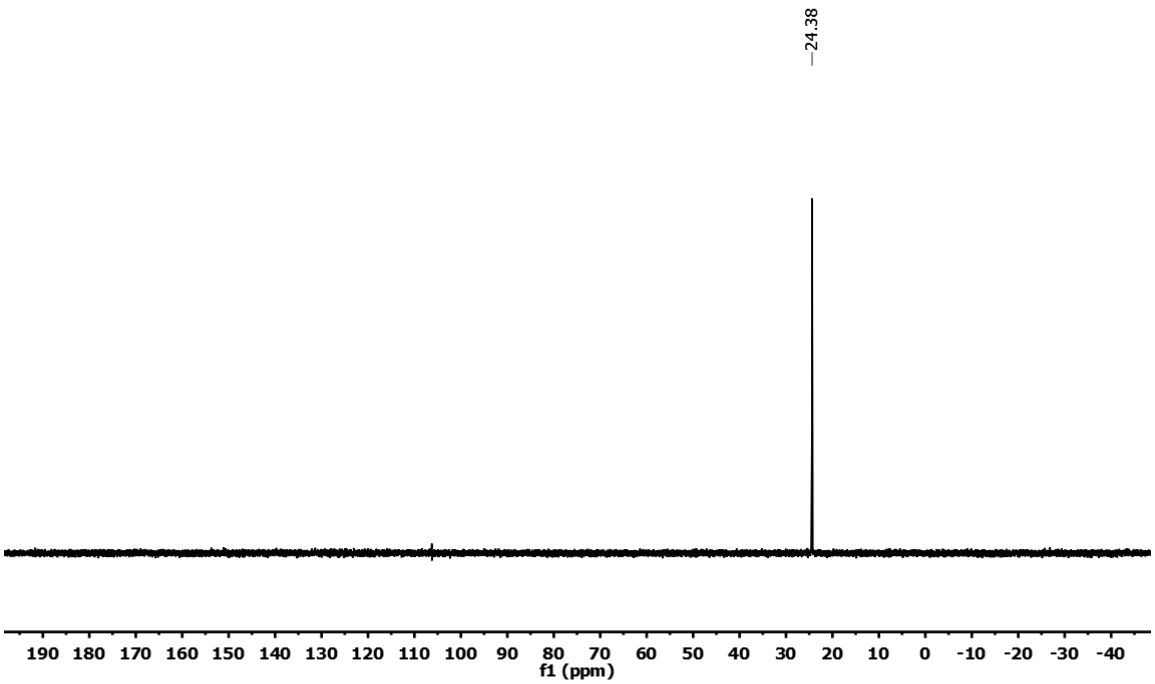


**Figure S17**. $^{31}P\{^{1}H\}$ NMR (202.31 MHz, 298 K, $CDCl_3$) of [Pd(C≡CPh)Cl(PN)] (**5**).

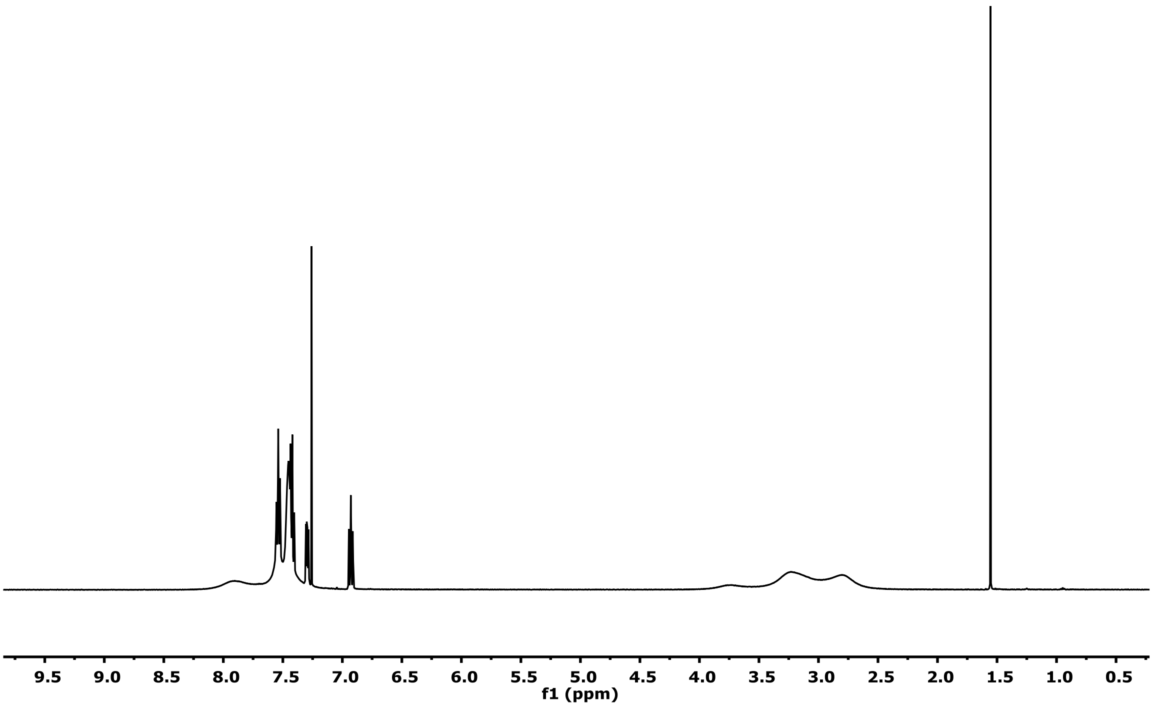


**Figure S18.** $^{1}$H NMR (499.72 MHz, 298 K, $CDCl_3$) of $[PdI_2(PN)]$.

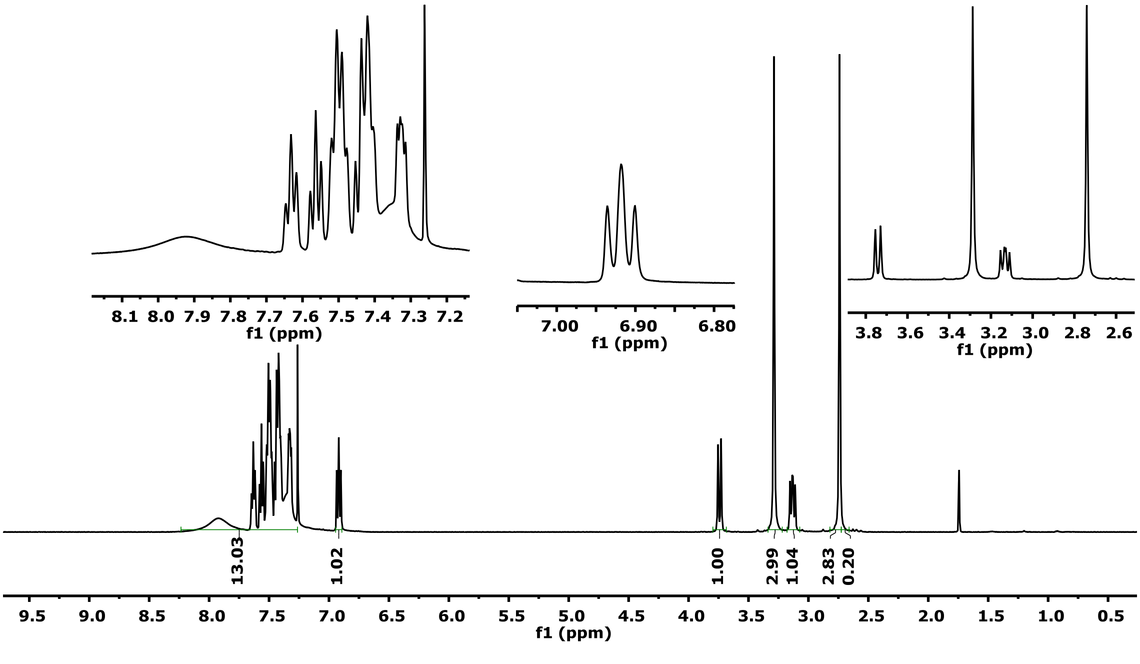


**Figure S19.** $^{1}$H NMR (499.72 MHz, 233 K, $CDCl_3$) of $[PdI_2(PN)]$.

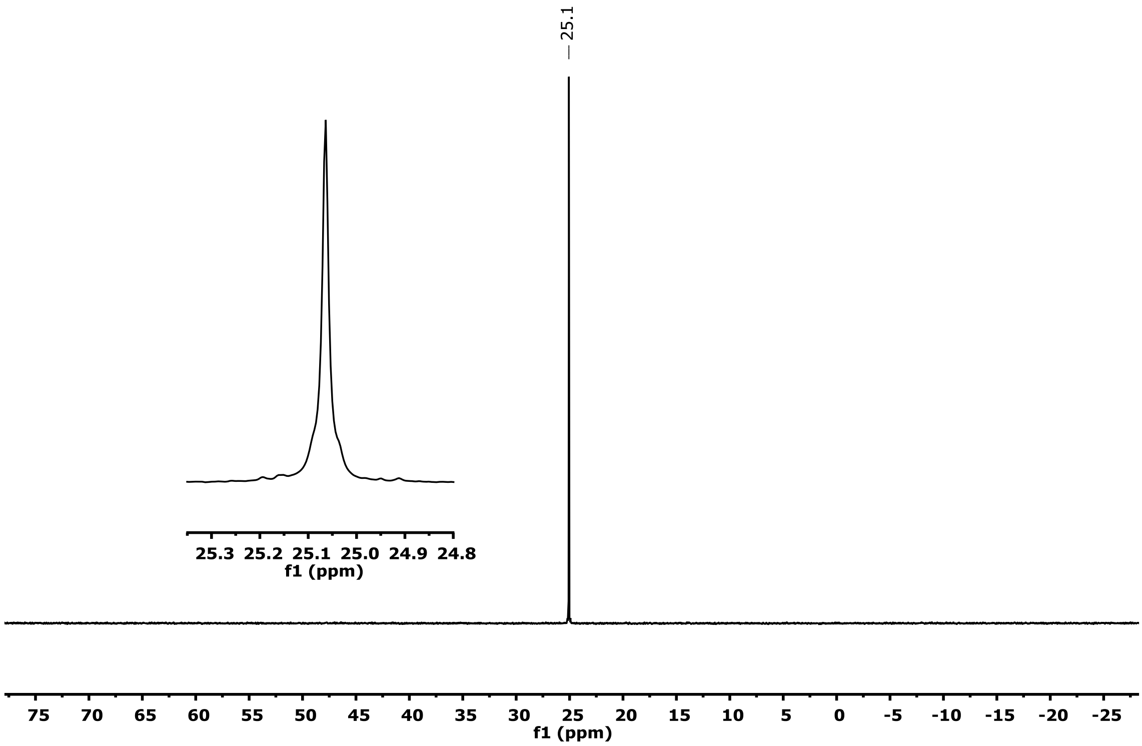


**Figure S20.** $^{31}P\{^{1}H\}$ NMR (202.31 MHz, 298 K, $CDCl_3$) of $[PdI_2(PN)]$.

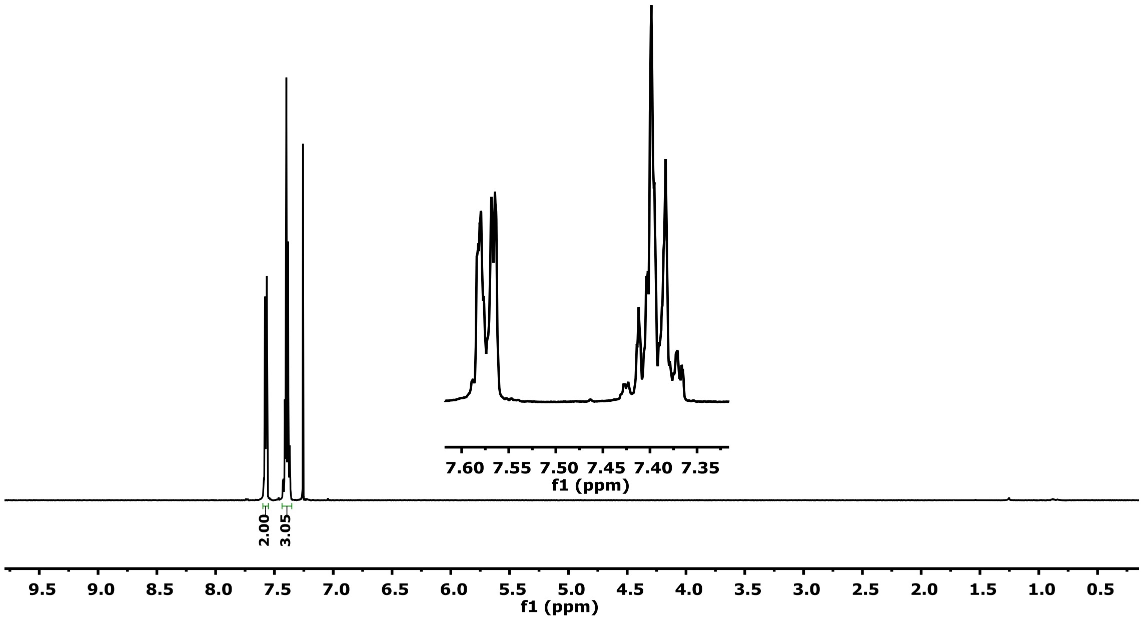


**Figure S21.** $^{1}H$ NMR (499.72 MHz, 298 K, $CDCl_3$) of $Ar^{F}$–C≡CPh.

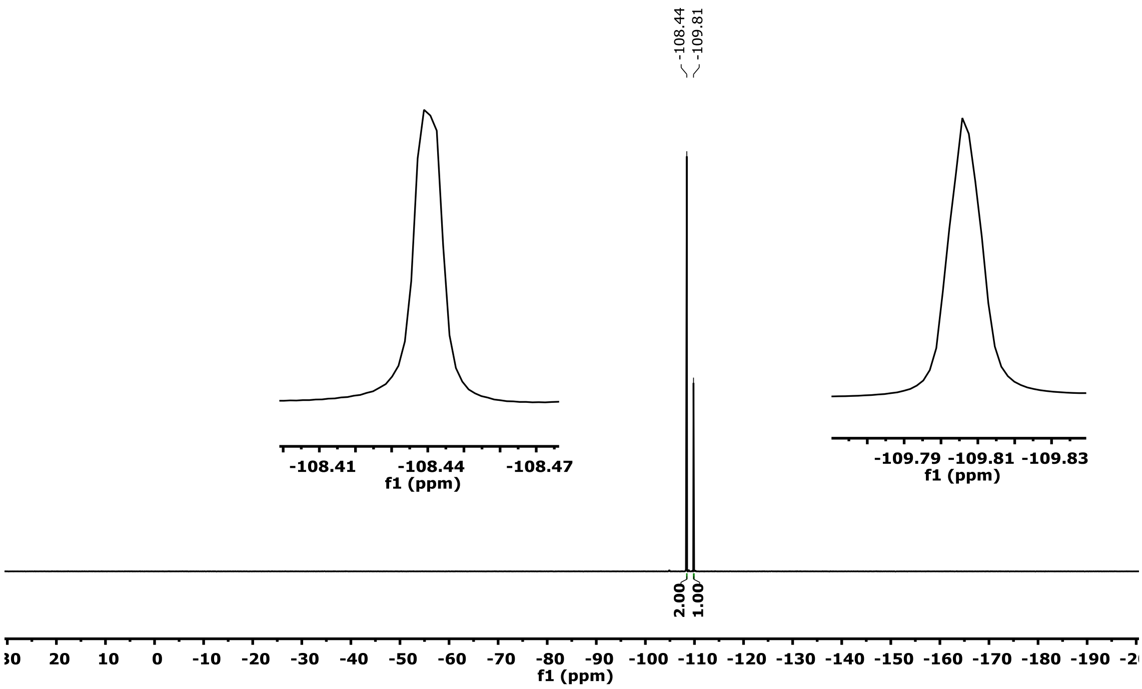


**Figure S22.** $^{19}F$ NMR (470.17 MHz, 298 K, $CDCl_3$) of $Ar^F$–C≡CPh.

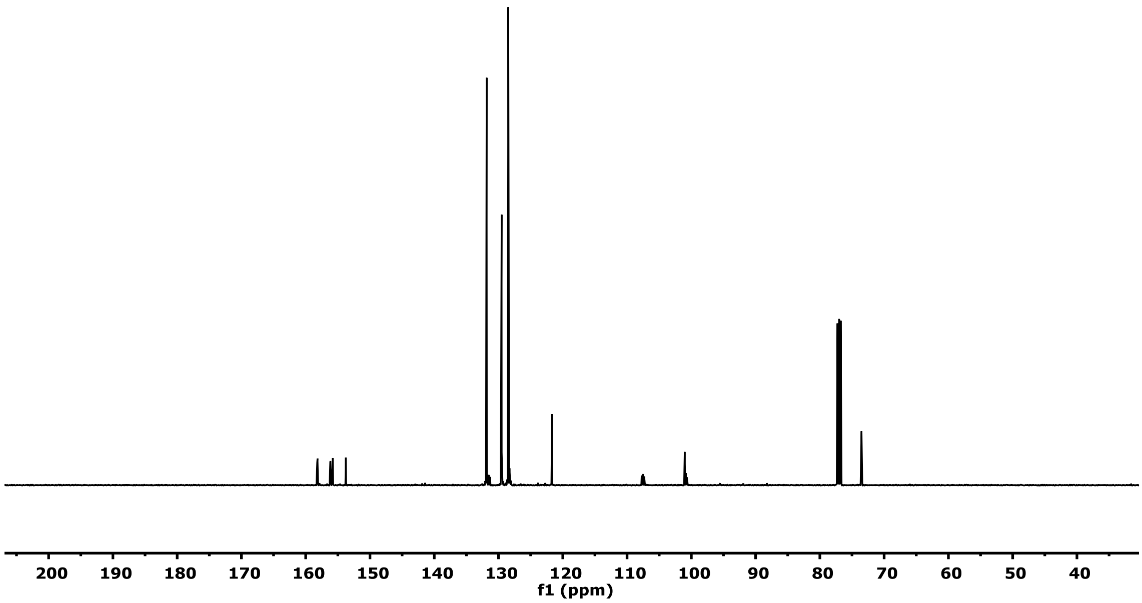


**Figure S23.** $^{13}C\{^1H\}$ NMR (125.67 MHz, 298 K, $CDCl_3$) of $Ar^F$–C≡CPh.

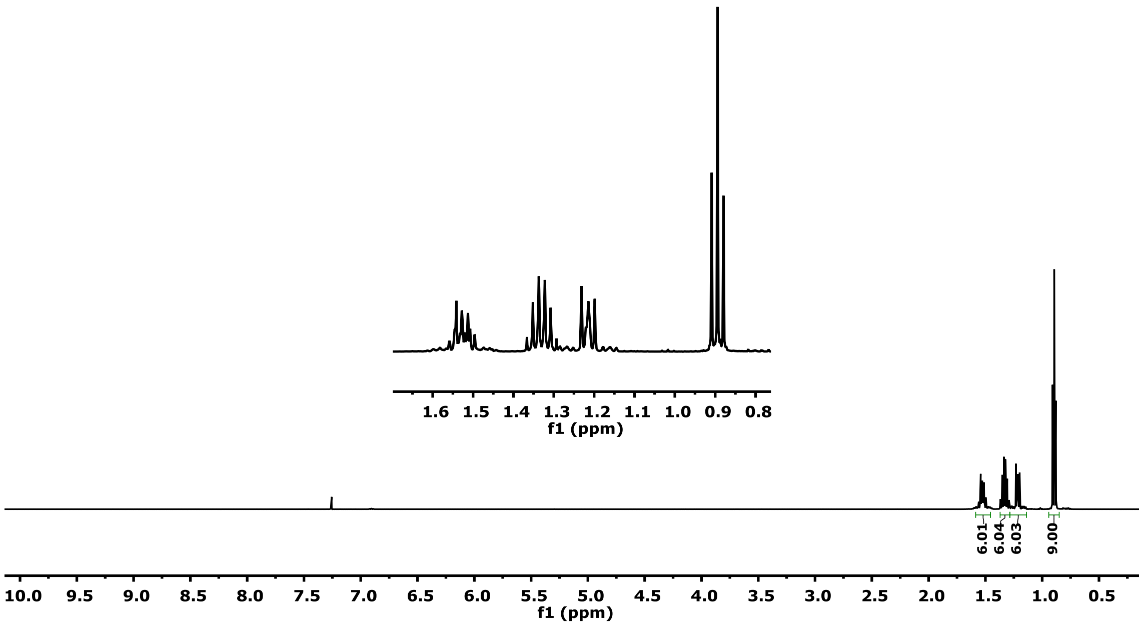


**Figure S24.** $^{1}$H NMR (499.72 MHz, 298 K, $CDCl_3$) of Ar$^{F}$–$SnBu_3$.

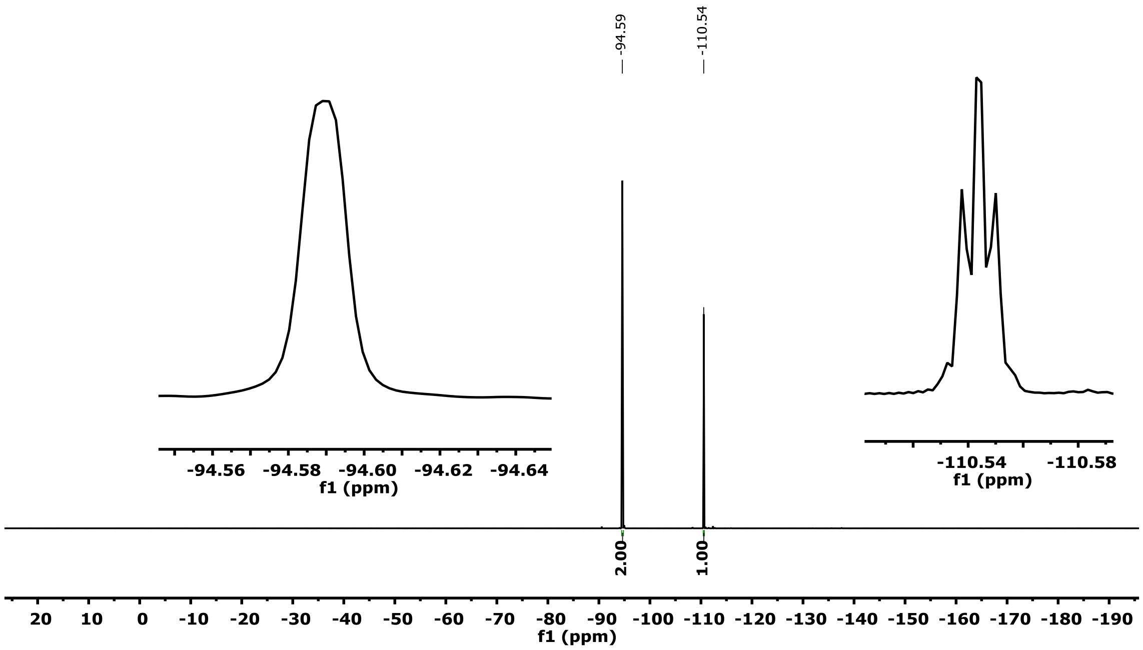


**Figure S25.** $^{19}$F NMR (470.17 MHz, 298 K, $CDCl_3$) of Ar$^{F}$–$SnBu_3$.

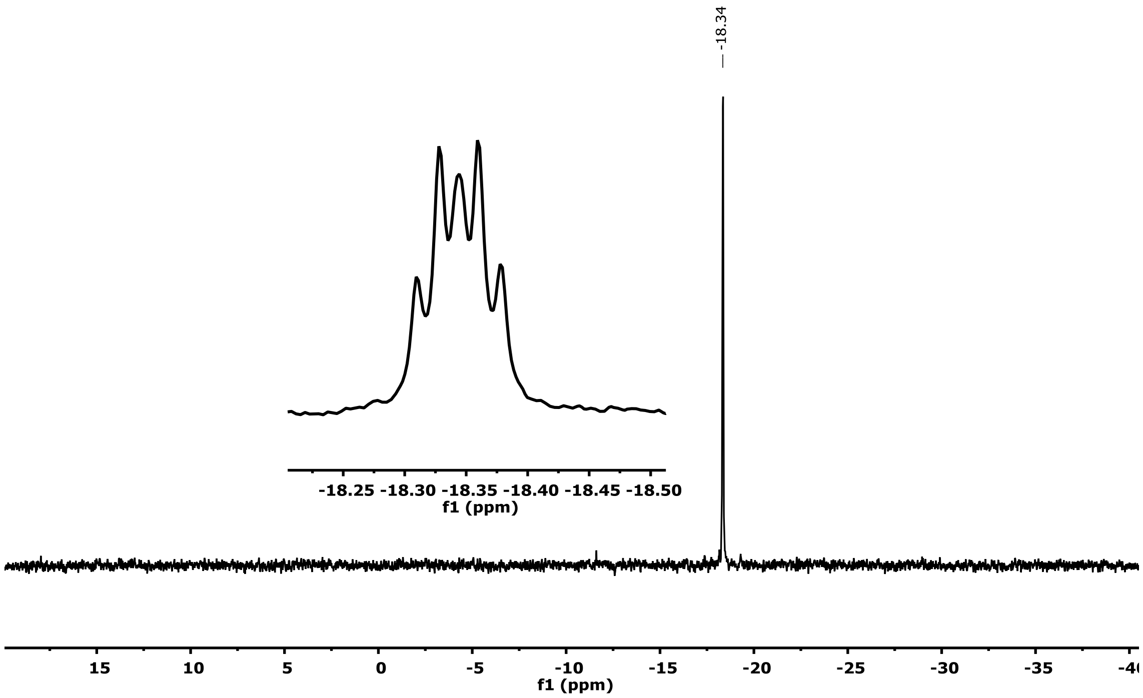


**Figure S26.** $^{119}Sn\{^{1}H\}$ NMR (186.35 MHz, 298 K, $CDCl_3$) of $Ar^F$–$SnBu_3$.

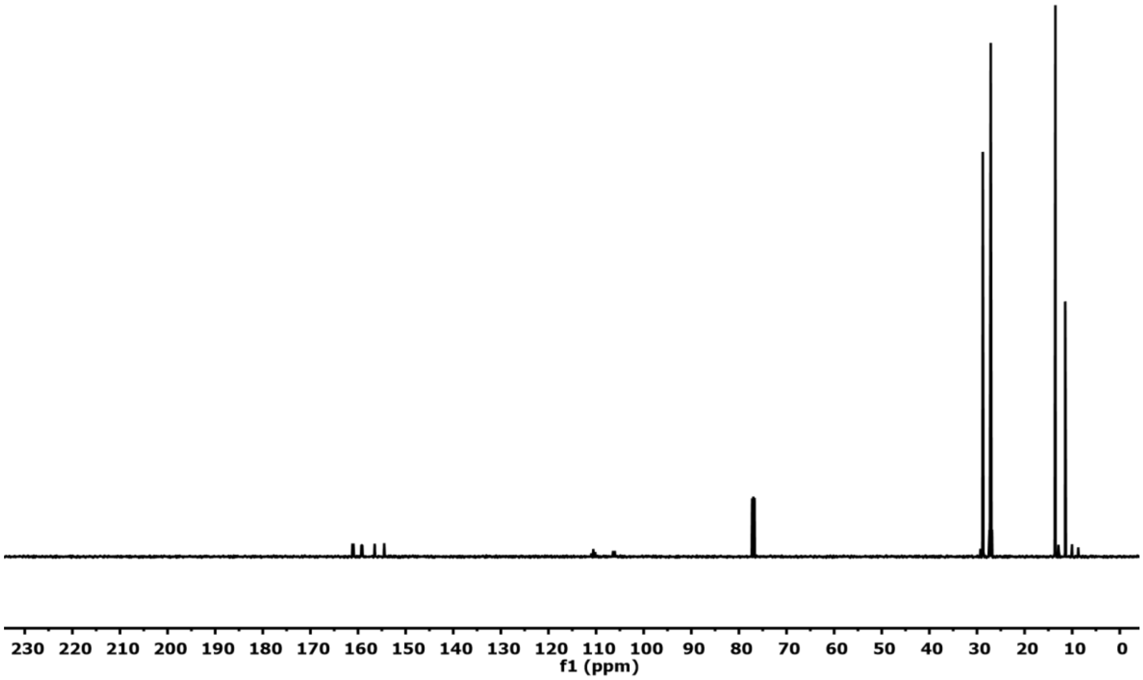


**Figure S27.** $^{13}C\{^{1}H\}$ NMR (125.67 MHz, 298 K, $CDCl_3$) of $Ar^F$–$SnBu_3$.

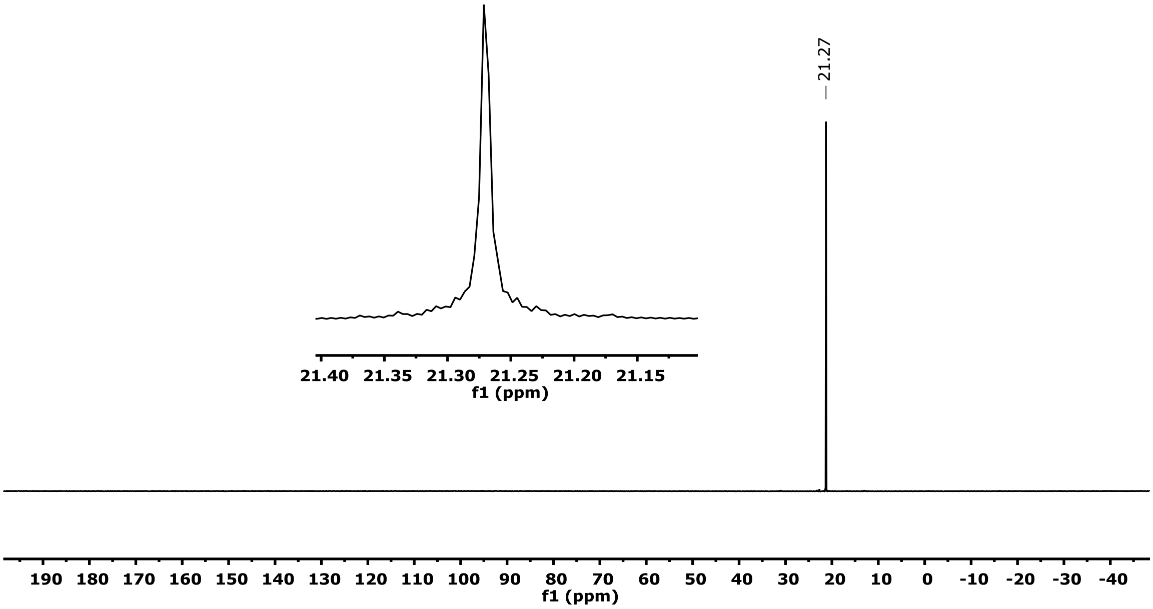


**Figure S28.** $^{31}P\{^{1}H\}$ NMR (202.31 MHz, 298 K, $CDCl_3$) of [Pd(PN)($\eta^2$-dmfu)].

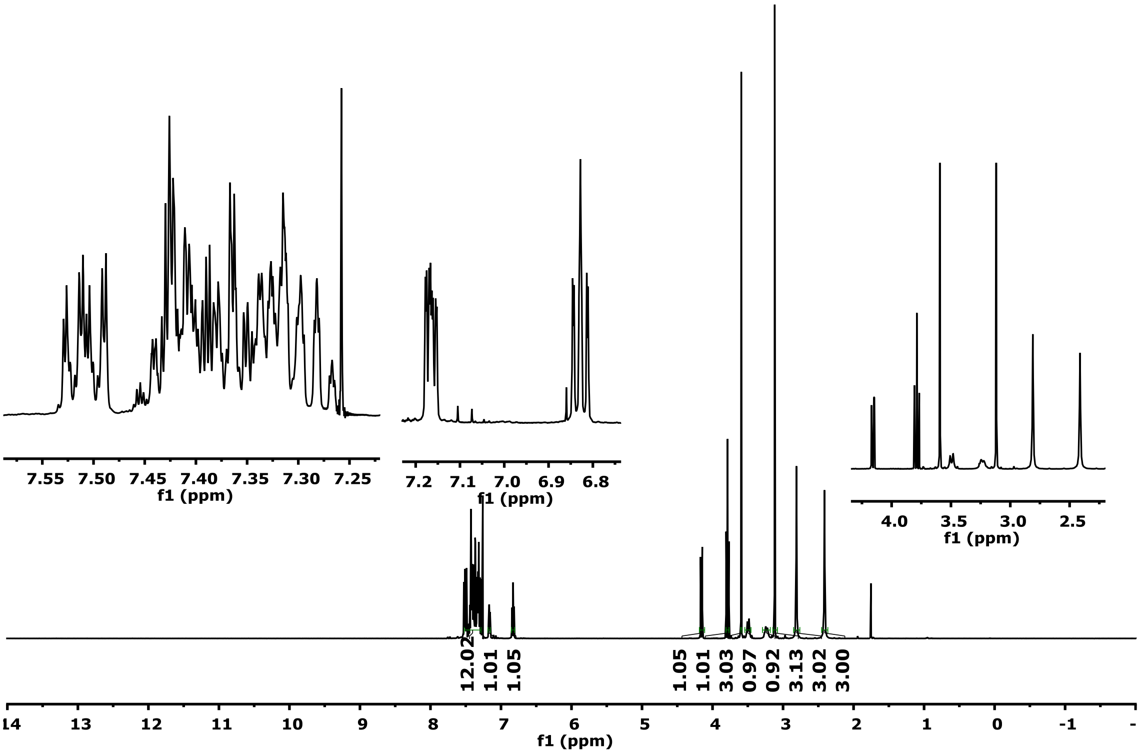


**Figure S29.** $^{1}H$ NMR (499.72 MHz, 298 K, $CDCl_3$) of [Pd(PN)($\eta^2$-dmfu)].

## 8. References

**1** Williams, D. B.; Lawton, M. Drying of organic solvents: quantitative evaluation of the efficiency of several desiccants. *J. Org. Chem.* **2010**, *75*, 8351–8354.

**2** Amman, C.; Meier, P.; Merbach, A. E. A simple multinuclear NMR thermometer. *J. Magn. Reson.* **1982**, *46*, 319–321.

**3** Tatsuno, Y.; Yoshida, T.; Otsuka. ($\eta^3$-Allyl)Palladium(II) Complexes. *Inorg. Synth.* **1990**, *28*, 342–345.

**4** Casares, J. A.; Espinet, P.; Martín-Alvarez, J. M.; Martínez-Ilarduya, J. M.; Salas, G. Stable Nickel Catalysts for Fast Norbornene Polymerization: Tuning Reactivity. *Eur. J. Inorg. Chem.* **2005**, 3825–3831.

**5** Rauchfuss, T. B.; Patino, F. T.; Roundhill, D. M. Platinum metal complexes of amine- and ether-substituted phosphines. *Inorg. Chem.* **1975**, *14*, 652–656.

**6** Casado, A. L.; Espinet, P. On the Configuration Resulting from Oxidative Addition of RX to $Pd(PPh_3)_4$ and the Mechanism of the cis-to-trans Isomerization of $[PdRX(PPh_3)_2]$ Complexes (R = Aryl, X = Halide). *Organometallics* **1998**, *17*, 954–959.

**7** Casado, A. L.; Espinet, P.; Gallego, A. M. Mechanism of the Stille Reaction. 2. Couplings of Aryl Triflates with Vinyltributyltin. Observation of Intermediates. A More Comprehensive Scheme. *J. Am. Chem. Soc.* **2000**, *122*, 11771–11782.

**8** Pérez-Temprano, M. H.; Gallego, A. M.; Casares, J. A.; Espinet, P. Stille Coupling of Alkynyl Stannane and Aryl Iodide, a Many-Pathways Reaction: The Importance of Isomerization. *Organometallics* **2011**, *30*, 611– 617.

**9** Gallego, A. M.; Peñas-Defrutos, M. N.; Marcos-Ayuso, G.; Martínez-Ilarduya, J. M.; Martín-Álvarez, J. M.; Espinet, P. Experimental study of speciation and mechanistic implications when using chelating ligands in aryl-alkynyl Stille coupling. *Dalton Trans.* **2020**, *49*, 11336–11345.

**10** Hartley, F. R.; Murray, S. G.; McAuliffe, C. A. Monomeric Complexes of Palladium(II) and Platinum(II) with a Series of Open-Chain Tetrathioether Ligands Prepared from Complexes of Weak Donor Ligands. *Inorg. Chem.* **1979**, *18*, 1394–1397.

**11** Dermenci, A.; Whittaker, R. E.; Gao, Y.; Cruz, F. A.; Yu, Z.-X.; Dong, G. Rh-catalyzed decarbonylation of conjugated ynones via carbon−alkyne bond activation: reaction scope and mechanistic exploration via DFT calculations. *Chem. Sci.* **2015**, *6*, 3201−3210.

**12** Usanov, D. L.: Yamamoto, H. Enantioselective Alkynylation of Aldehydes with 1-Haloalkynes Catalyzed by Tethered Bis(8-quinolinato) Chromium Complex. *J. Am. Chem. Soc.* **2011**, *133*, 1286–1289.

**13** Product Subclass 1: 1-Haloalk-1-ynes and Alk-1-yn-1-ols. Witulski, B.; Alayrac, C., *Science of Synthesis*, **2006**, *24*, 905.

**14** Roesler, F.; Kovács, M.; Bruhn, C.; Kelemen, Z.; Pietschnig, R. Phosphetes via Transition Metal Free Ring Closure–Takingthe Proper Turn at a Thermodynamic Crossing. *Chem. Eur. J.* **2021**, *27*, 9782–9790.

**15** Wei, Y.; Zhao, H.; Kan, J.; Su, W.; Hong, M. Copper-Catalyzed Direct Alkynylation of Electron-Deficient Polyfluoroarenes with Terminal Alkynes Using O2 as an Oxidant. *J. Am. Chem. Soc.* **2010**, *132*, 2522−2523.

**16** Gioria, E.; Martínez-Ilarduya, J. M.; García-Cuadrado, D.; Miguel, J. A.; Genov, M.; Espinet, P. Phosphines with Tethered Electron-Withdrawing Olefins as Ligands for Efficient Pd-Catalyzed Aryl-Alkyl Coupling. *Organometallics* **2013**, *32*, 4255−4261.

**17** Dermenci, A.; Whittaker, R. E.; Gao, Y.; Cruz, F. A.; Yu, Z.-X.; Dong, G. Rh-catalyzed decarbonylation of conjugated ynones via carbon−alkyne bond activation: reaction scope and mechanistic exploration via DFT calculations. *Chem. Sci*. **2015**, *6*, 3201−3210.

**18** Bizier, N. P.; Wackerly, J. W.; Braunstein, E. D.; Zhang, M.; Nodder, S. T.; Carlin, S. M.; Katz, J. L. An Alternative Role for Acetylenes: Activation of Fluorobenzenes toward Nucleophilic Aromatic Substitution. *J. Org. Chem*. **2013**, *78*, 5987−5998.

**19** Compound previously reported in: Fierro-Arias, J. G.; Redon, R.; Garcia, J. J.; Hernandez-Ortega, S.; Toscano, R. A.; Morales-Morales, D. Pd catalyzed Heck reaction with the catalytic system [Pd($Ph_2PC_6H_4$-2-($CH_2NMe_2$))($SRF)_2$]: Examination of the electronic effects of fluorinated thiolates. *J. Mol. Cat. A* **2005**, *233*, 17–27.

**20** Hoops, S.; Sahle, S.; Gauges, R.; Lee, C.; Pahle, J.; Simus, N.; Singhal, M.; Xu, L.; Mendes, P.; Kummer, U. COPASI—a COmplex PAthway SImulator. *Bioinformatics* **2006**, *22*, 3067–3074.

**21** Chai, J. D.; Head-Gordon, M. Long-Range Corrected Hybrid Density Functionals with Damped Atom-Atom Dispersion Corrections. *Phys. Chem. Chem. Phys*. **2008**, *10*, 6615–6620.

**22** Gaussian 09, Revision D.01, Frisch, M. J.; Trucks, G. W.; Schlegel, H. B.; Scuseria, G. E.; Robb, M. A.; Cheeseman, J. R.; Scalmani, G.; Barone, V.; Mennucci, B.; Petersson, G. A.; Nakatsuji, H.; Caricato, M.; Li, X.; Hratchian, H. P.; Izmaylov, A. F.; Bloino, J.; Zheng, G.; Sonnenberg, J. L.; Hada, M.; Ehara, M.; Toyota, K.; Fukuda, R.; Hasegawa, J.; Ishida, M.; Nakajima, T.; Honda, Y.; Kitao, O.; Nakai, H.; Vreven, T.; Montgomery, J. A., Jr.; Peralta, J. E.; Ogliaro, F.; Bearpark, M.; Heyd, J. J.; Brothers, E.; Kudin, K. N.; Staroverov, V. N.; Kobayashi, R.; Normand, J.; Raghavachari, K.; Rendell, A.; Burant, J. C.; Iyengar, S. S.; Tomasi, J.; Cossi, M.; Rega, N.; Millam, M. J.; Klene, M.; Knox, J. E.; Cross, J. B.; Bakken, V.; Adamo, C.; Jaramillo, J.; Gomperts, R.; Stratmann, R. E.; Yazyev, O.; Austin, A. J.; Cammi, R.; Pomelli, C.; Ochterski, J. W.; Martin, R. L.; Morokuma, K.; Zakrzewski, V. G.; Voth, G. A.; Salvador, P.; Dannenberg, J. J.; Dapprich, S.; Daniels, A. D.; Farkas, Ö.; Foresman, J. B.; Ortiz, J. V.; Cioslowski, J.; Fox, D. J. Gaussian, Inc., Wallingford CT, **2009**.

**23** Carrasco, D.; García-Melchor, M.; Casares, J. A.; Espinet, P. Dramatic mechanistic switch in Sn/AuI group exchanges: transmetalation vs. oxidative addition. *Chem. Commun*. **2016**, *52*, 4305–4308.

**24** (a) Hay, P. J.; Wadt, W. R. Ab Initio Effective Core Potentials for Molecular Calculations. Potentials for the Transition Metal Atoms Sc to Hg. *J. Chem. Phys*. **1985**, *82*, 270–283. (b) Hay, P. J.; Wadt, W. R. Ab Initio Effective Core Potentials for Molecular Calculations. Potentials for K to Au Including the Outermost Core Orbitale. *J. Chem. Phys*. **1985**, *82*, 299–310.

**25** Ehlers, A. W.; Böhme, M.; Dapprich, S.; Gobbi, A.; Höllwarth, A.; Jonas, V.; Köhler, K. F.; Stegmann, R.; Veldkamp, A.; Frenking, G. A set of f-polarization functions for pseudo-potential basis sets of the transition metals Sc-Cu, Y-Ag and La-Au. *Chem. Phys. Lett*. **1993**, *208*, 111–114.

**26** Ehlers, A. W.; Böhme, M.; Dapprich, S.; Gobbi, A.; Höllwarth, A.; Jonas, V.; Köhler, K. F.; Stegmann, R.; Veldkamp, A.; Frenking, G. A set of d-polarization functions for pseudo-potential basis sets of the main group elements Al-Bi and f-type polarization functions for Zn, Cd, Hg. *Chem. Phys. Lett.* **1993**, *208*, 237–240.

**27** Marenich, A. V.; Cramer, C. J.; Truhlar, D. G. Universal Solvation Model Based on Solute Electron Density and on a Continuum Model of the Solvent Defined by the Bulk Dielectric Constant and Atomic Surface Tensions. *J. Phys. Chem. B* **2009**, *113*, 6378–6396.

**28** Moncarz, J. R.; Brunker, T. J.; Jewett, J. C.; Orchowski, M.; Glueck, D. S.; Sommer, R. D.; Lam, K.-C.; Incarvito, C. D.; Concolino, T. E.; Ceccarelli, C.; Zakharov, L. N.; Rheingold, A. L. Palladium-Catalyzed Asymmetric Phosphination. Enantioselective Synthesis of PAMP−$BH_3$, Ligand Effects on Catalysis, and Direct Observation of the Stereochemistry of Transmetalation and Reductive Elimination. *Organometallics* **2003**, *22*, 3205–3221.

**29** Compound previously reported in: Toledo, A.; Funes-Ardoiz, I.; Maseras, F.; Albéniz, A. C. Palladium-Catalyzed Aerobic Homocoupling of Alkynes: Full Mechanistic Characterization of a More Complex Oxidase-Type Behavior. *ACS Catal*. **2018**, *8*, 7495–7506.

**30** Ye, X.; Zhao, P.; Zhang, S.; Zhang, Y.; Wang, Q.; Shan, C.; Wojtas, L.; Guo, H.; Chen, H.; Shi, X. Facilitating Gold Redox Catalysis with Electrochemistry: An Efficient Chemical-Oxidant-Free Approach. *Angew. Chem., Int. Ed*. **2019**, *58*, 17226−17230.

**31** CrysAlisPro Software system, version 1.171.33.51, 2009, Oxford Diffraction Ltd, Oxford, UK.

**32** Dolomanov, O. V.; Bourhis, L. J.; Gildea, R. J.; Howard, J. A. K.; Puschmann, H. OLEX2: a complete structure solution, refinement and analysis program. *J. Appl. Cryst.* **2009**, *42*, 339–341.

**33** Sheldrick, G. M. SHELXT - Integrated space-group and crystal-structure determination. *Acta Cryst.* **2015**, *A71*, 3–8.

**34** Sheldrick, G. M. Crystal structure refinement with SHELXL. *Acta Cryst*. **2015**, *C71*, 3–8.
